# Supplementary figures and images for: Radiotherapy to the prostate for men with metastatic prostate cancer in the UK and Switzerland: Long-term results from the STAMPEDE randomised controlled trial
Source: PLoS Med. 2022 Jun 7;19(6):e1003998. doi: 10.1371/journal.pmed.1003998 (PMC9173627; doi:10.1371/journal.pmed.1003998)

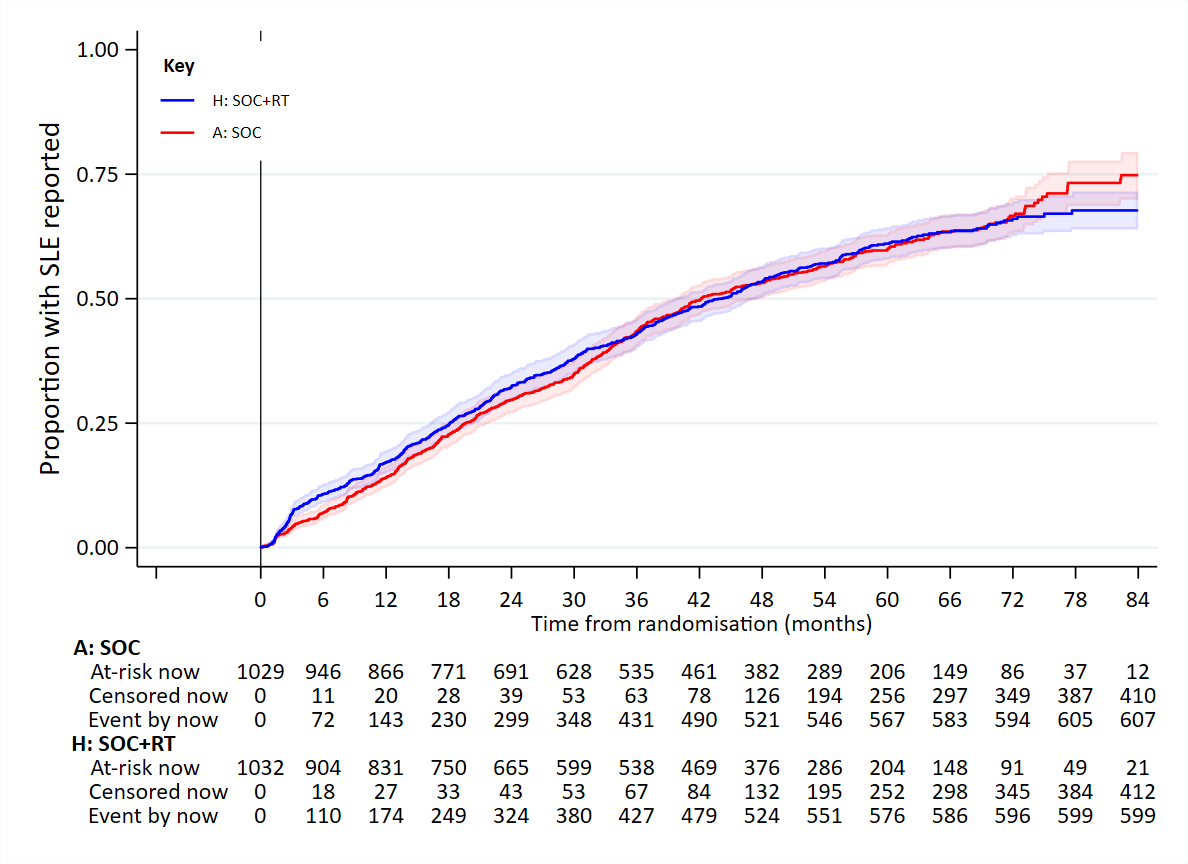

Supplement: S1 Fig — Adjusted HR = 1.00 (95% CI 0.90 to 1.13; p = 0.931). HR, hazard ratio; RT, radiotherapy to the prostate; SLE, symptomatic local event; SOC, standard of care. (TIF) [file pmed.1003998.s001.tif]

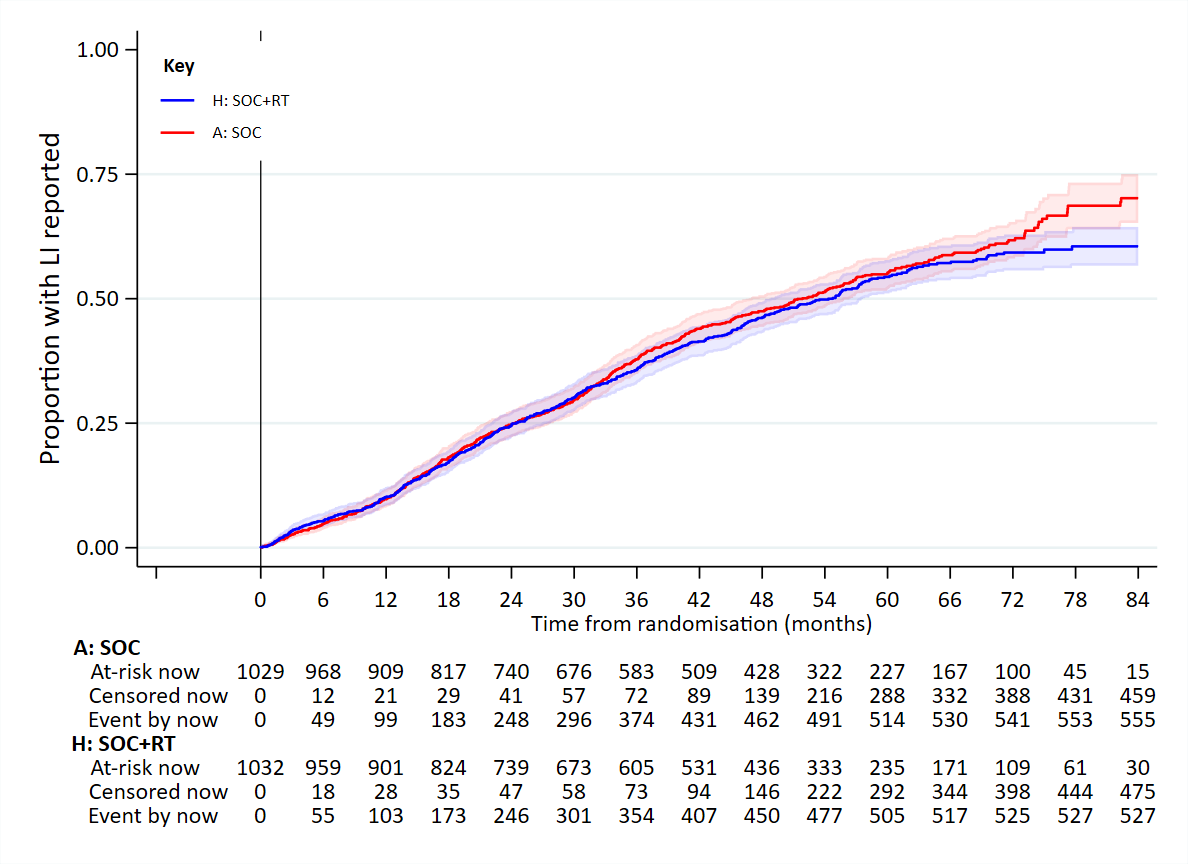

Supplement: S2 Fig — Adjusted HR = 0.94 (95% CI 0.83 to 1.06; p = 0.286). HR, hazard ratio; LI, local intervention; RT, radiotherapy to the prostate; SOC, standard of care. (TIF) [file pmed.1003998.s002.tif]

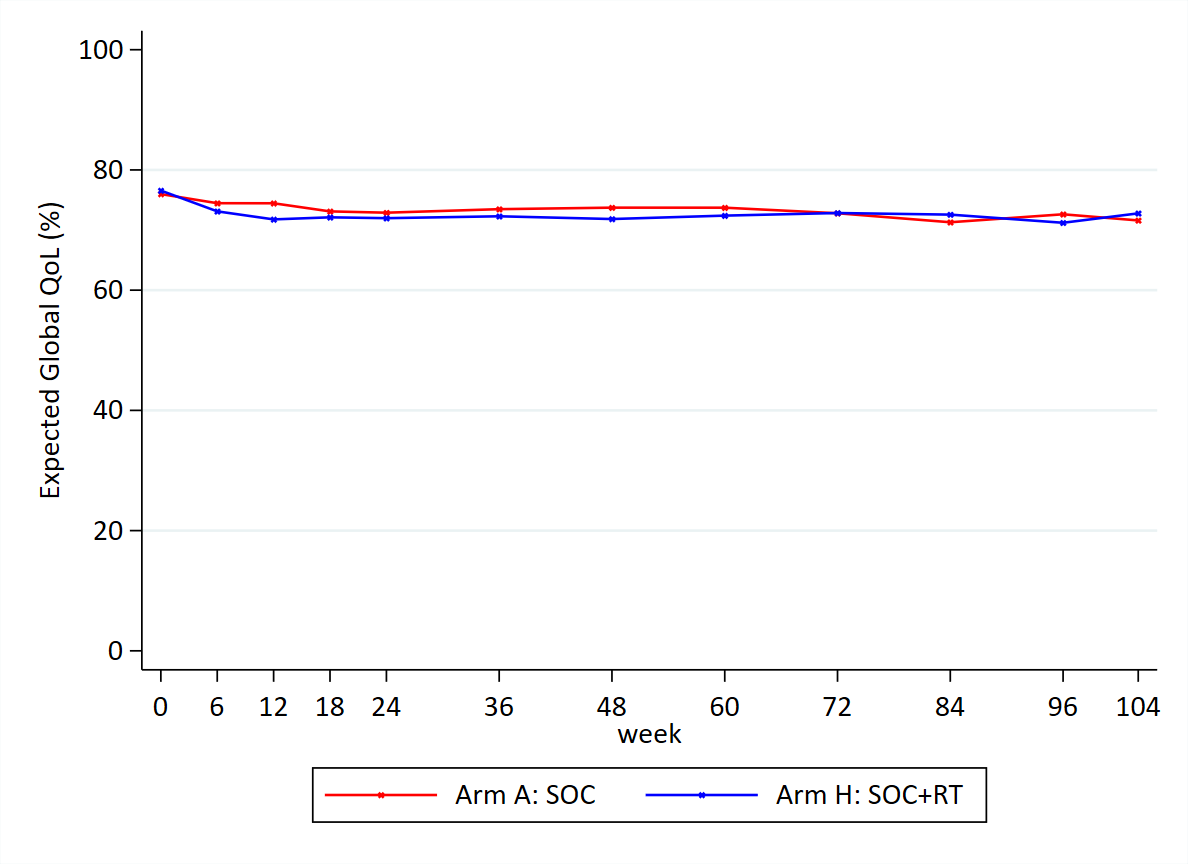

Supplement: S3 Fig — Difference in weighted average: −0.8% (95% CI −2.5% to 0.9%; p = 0.349). QoL, quality of life; RT, radiotherapy to the prostate; SOC, standard of care. (TIF) [file pmed.1003998.s003.tif]

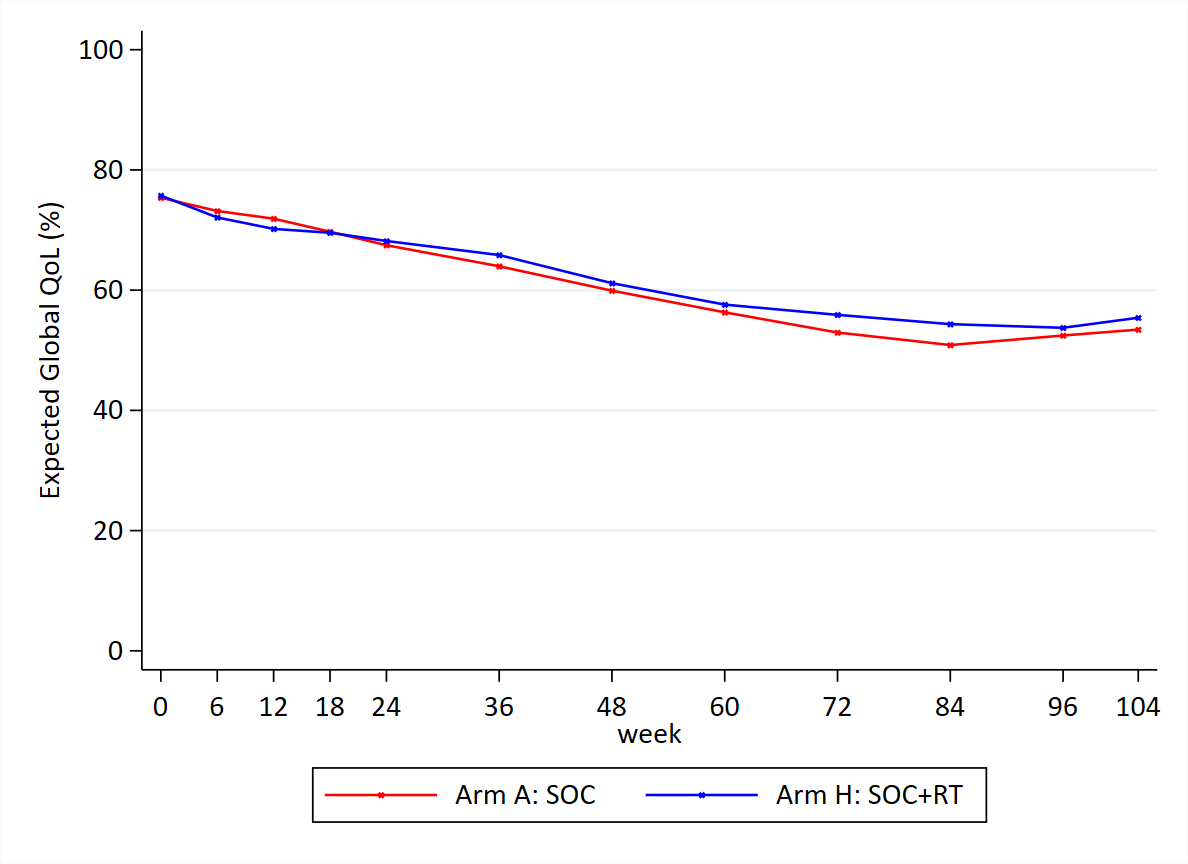

Supplement: S4 Fig — Difference in weighted average: 1.3% (95% CI −1.1% to 3.8%; p = 0.287). QoL, quality of life; RT, radiotherapy to the prostate; SOC, standard of care. (TIF) [file pmed.1003998.s004.tif]

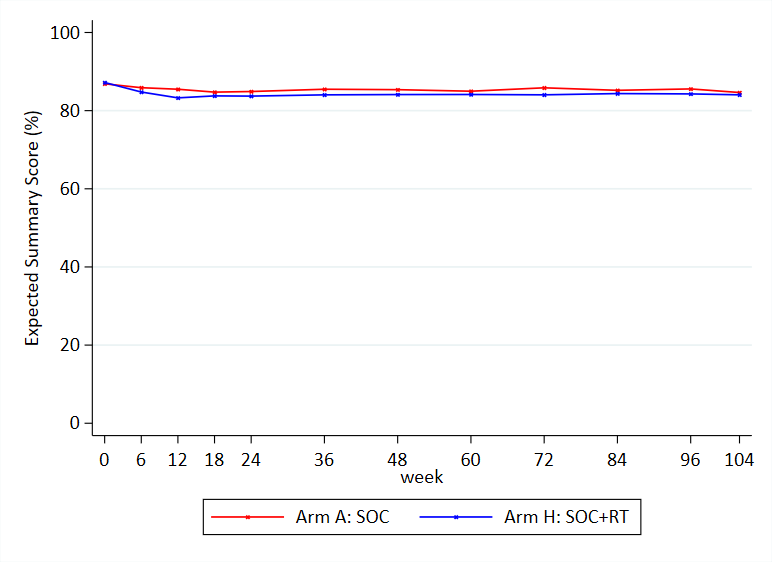

Supplement: S5 Fig — Difference in weighted average: −1.2% (95% CI −2.4% to 0.0%; p = 0.050). RT, radiotherapy to the prostate; SOC, standard of care. (TIF) [file pmed.1003998.s005.tif]

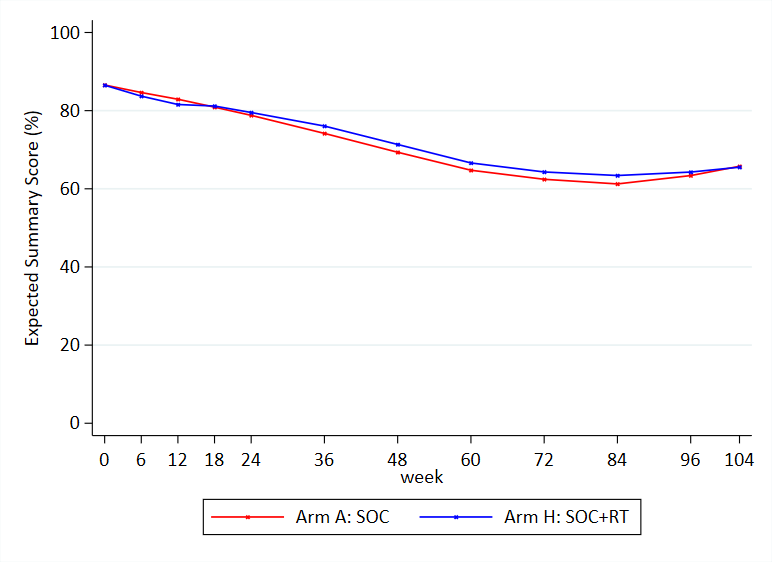

Supplement: S6 Fig — Difference in weighted average: 1.2% (95% CI −1.3% to 3.6%; p = 0.365). RT, radiotherapy to the prostate; SOC, standard of care. (TIF) [file pmed.1003998.s006.tif]

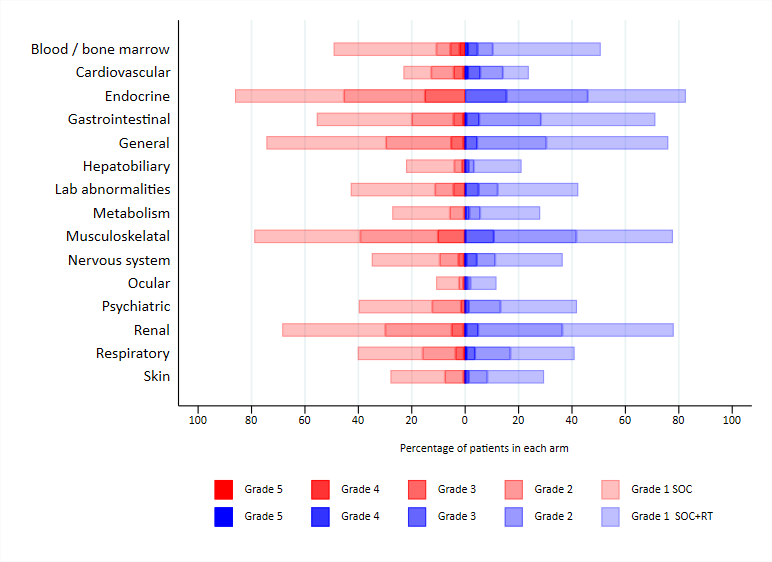

Supplement: S7 Fig — AE, adverse event; CTCAE, Common Terminology Criteria for Adverse Events; RT, radiotherapy to the prostate; SOC, standard of care. (TIF) [file pmed.1003998.s007.tif]

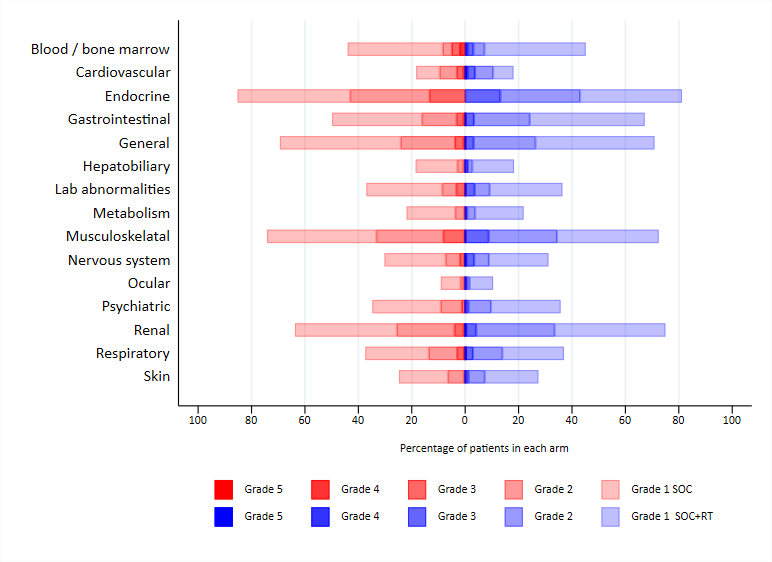

Supplement: S8 Fig — AE, adverse event; CTCAE, Common Terminology Criteria for Adverse Events; RT, radiotherapy to the prostate; SOC, standard of care. (TIF) [file pmed.1003998.s008.tif]

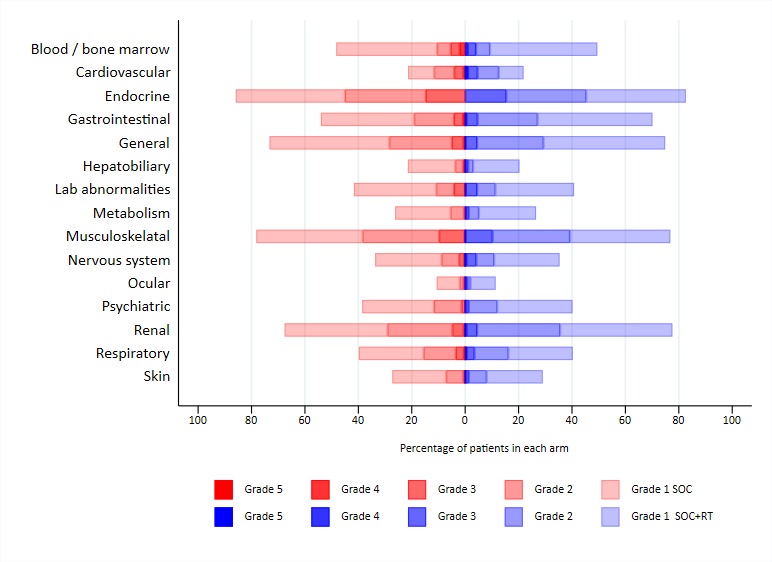

Supplement: S9 Fig — AE, adverse event; CTCAE, Common Terminology Criteria for Adverse Events; RT, radiotherapy to the prostate; SOC, standard of care. (TIF) [file pmed.1003998.s009.tif]

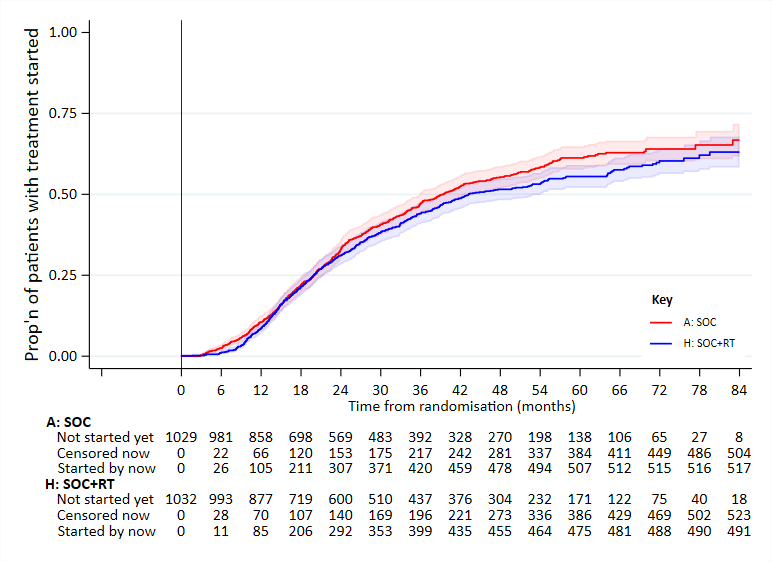

Supplement: S10 Fig — RT, radiotherapy to the prostate; SOC, standard of care. (TIF) [file pmed.1003998.s010.tif]

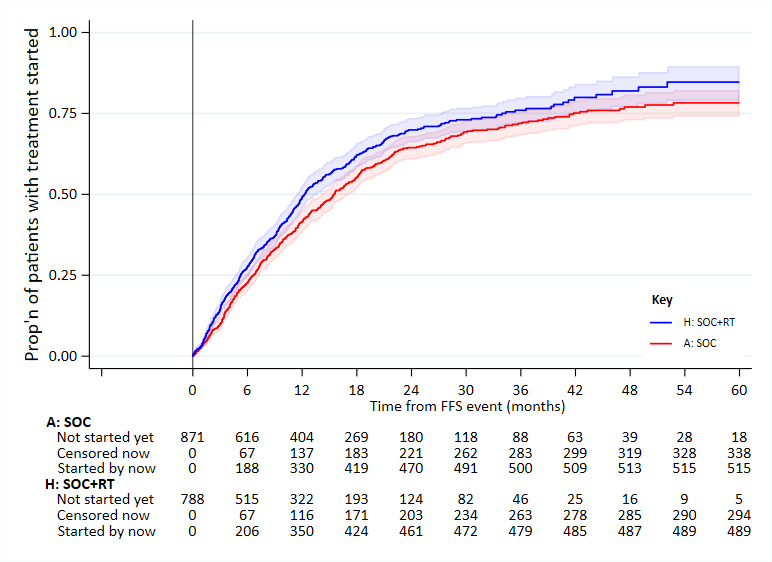

Supplement: S11 Fig — FFS, failure-free survival; RT, radiotherapy to the prostate; SOC, standard of care. (TIF) [file pmed.1003998.s011.tif]
