# Supplementary material for: Radiotherapy to the prostate for men with metastatic prostate cancer in the UK and Switzerland: Long-term results from the STAMPEDE randomised controlled trial
Source: PLoS Med. 2022 Jun 7;19(6):e1003998. doi: 10.1371/journal.pmed.1003998 (PMC9173627; doi:10.1371/journal.pmed.1003998)
Supplement: S1 Text — ADT, androgen deprivation therapy; IQR, interquartile range; PSA, prostate specific antigen; RT, radiotherapy to the prostate; SOC, standard of care; WHO, World Health Organization. Table B in S1 Text. Eligibility status following participant audit. RT, radiotherapy to the prostate; SOC, standard of care. Table C in S1 Text. Sensitivity analyses on OS based on explicit eligibility. ITT, intention-to-treat; OS, overall survival; RT, radiotherapy to the prostate; SOC, standard of care. Table D in S1 Text. First local intervention event reported (patients with event reported). PCa, prostate cancer; RT, radiotherapy to the prostate; SOC, standard of care; TURP, transurethral resection of the prostate. Table E in S1 Text. Summary of analyses of time to local event outcomes. *Subdistribution HR for competing risks models. ^Cox model, adjusting for age, nodal involvement, WHO performance status, regular aspirin or NSAID use and planned SOC docetaxel at randomisation, stratified by randomisation time period. +Fine and Gray model with outcome excluding PCa death and death from any cause as competing risk. NSAID, nonsteroidal anti-inflammatory drug; PCa, prostate cancer; SOC, standard of care; WHO, World Health Organization. Table F in S1 Text. Grade 3 to 5 late RT toxicities reported over entire time on trial (RTOG). Note: Treatment arms correspond to safety population; patients with ≥1 Follow-Up CRF returned. RT, radiotherapy to the prostate; RTOG, Radiation Therapy Oncology Group; SOC, standard of care. Table G in S1 Text. Grade 3 to 5 late RT toxicities reported at 2 years (RTOG). Note: Treatment arms correspond to safety population; patients with ≥1 Follow-Up CRF returned and no reported progression at 2 years. RT, radiotherapy to the prostate; RTOG, Radiation Therapy Oncology Group; SOC, standard of care. Table H in S1 Text. Grade 3 to 5 late RT toxicities reported at 4 years (RTOG). Note: Treatment arms correspond to safety population; patients with ≥1 Follow-Up CRF re [file pmed.1003998.s012.docx]

# S1 TexT: SUPPLEMENTARY TABLES

Table A: Baseline Characteristics For Metastatic Volume Analyses

| **Characteristic** |  | **SOC** | | **SOC+RT** | |
| --- | --- | --- | --- | --- | --- |
|  |  | Lower metastatic burden  (n=409) | Higher metastatic burden  (n=567) | Lower metastatic burden  (n=410) | Higher metastatic burden  (n=553) |
| Age at randomisation (years) | Median (IQR) | 68 (63-73) | 68 (63-73) | 68 (63-73) | 68 (63-73) |
|  | Range | 44-83 | 37-86 | 45-84 | 46-87 |
|  |  |  |  |  |  |
| WHO Performance Status | 0 | 305 (75%) | 390 (69%) | 313 (76%) | 376 (68%) |
|  | 1-2 | 104 (25%) | 177 (31%) | 97 (24%) | 177 (32%) |
|  |  |  |  |  |  |
| Pain from prostate cancer | Absent | 366 (90%) | 423 (75%) | 367 (90%) | 442 (80%) |
|  | Present | 42 (10%) | 141 (25%) | 40 (10%) | 109 (20%) |
|  | *Missing* | 1 | 3 | 3 | 2 |
|  |  |  |  |  |  |
| Previous notable health  issues* | Myocardial infarction | *25 (6%)* | *35 (6%)* | 20 (5%) | 37 (7%) |
|  | Cerebrovascular disease | 14 (3%) | 13 (2%) | 10 (2%) | 18 (3%) |
|  | Congestive heart failure | 5 (1%) | 0 (0%) | 3 (1%) | 5 (1%) |
|  | Angina | 23 (6%) | 21 (4%) | 19 (5%) | 31 (6%) |
|  | Hypertension | 168 (41%) | 222 (39%) | 181 (44%) | 240 (43%) |
|  |  |  |  |  |  |
| T-category at randomisation | T0 | 0 (0%) | 0 (0%) | 1 (<1%) | 0 (0%) |
|  | T1 | 7 (2%) | 4 (1%) | 6 (2%) | 5 (1%) |
|  | T2 | 39 (10%) | 39 (8%) | 32 (8%) | 50 (10%) |
|  | T3 | 250 (64%) | 305 (61%) | 261 (66%) | 302 (61%) |
|  | T4 | 95 (24%) | 151 (30%) | 94 (24%) | 138 (28%) |
|  | TX | 18 | 68 | 16 | 58 |
|  |  |  |  |  |  |
| N-category at randomisation | N0 | 144 (37%) | 188 (36%) | 142 (36%) | 187 (38%) |
|  | N+ | 249 (63%) | 333 (64%) | 258 (65%) | 311 (62%) |
|  | NX | 16 | 46 | 10 | 55 |
|  |  |  |  |  |  |
| Sites of metastases | Bone | 311 (76%) | 561 (99%) | 311 (76%) | 549 (99%) |
|  | Liver | 0 (0%) | 22 (4%) | 0 (0%) | 18 (3%) |
|  | Lung | 0 (0%) | 36 (6%) | 0 (0%) | 41 (7%) |
|  | Distant lymph nodes | 140 (34%) | 138 (24%) | 149 (36%) | 128 (23%) |
|  | Other | 18 (4%) | 16 (3%) | 16 (4%) | 15 (3%) |
|  |  |  |  |  |  |
| Gleason sum score | <=7 | 77 (19%) | 84 (15%) | 86 (22%) | 82 (15%) |
|  | 8-10 | 326 (81%) | 460 (85%) | 311 (78%) | 454 (85%) |
|  | Unknown | 6 | 23 | 13 | 17 |
| PSA pre-ADT (ng/ml) | Median (IQR) | 48 (19-120) | 181 (60-619) | 55 (23-138) | 180 (52-668) |
|  | Range | 2-5560 | 1-20590 | 1-1706 | 2-11156 |
|  |  |  |  |  |  |
| Time from diagnosis (Days) | Median (IQR) | 81 (63-103) | 69 (49-86) | 80 (59-101) | 69 (51-88) |
|  | Range | 6-2297 | 0-3495 | 9-1276 | 0-821 |
|  | *Missing* | 0 | 1 | 0 | 2 |
|  |  |  |  |  |  |
| Days from starting hormones | Median (IQR) | 49 (32-68) | 55 (38-71) | 52 (32-69) | 58 (37-73) |
|  | Range | -3;84 | -1;84 | 0;86 | 0;84 |
|  | *Missing* | 9 | 8 | 7 | 4 |
|  |  |  |  |  |  |
| Planned SOC docetaxel | No | 342 (84%) | 462 (81%) | 348 (85%) | 444 (80%) |
|  | Yes | 67 (16%) | 105 (19%) | 62 (15%) | 109 (20%) |
|  |  |  |  |  |  |
| Nominated RT schedule | 36Gy/6f/6wk | 190 (46%) | 257 (45%) | 170 (41%) | 290 (52%) |
|  | 55Gy/20f/4wk | 219 (54%) | 310 (55%) | 240 (59%) | 263 (48%) |
|  |  |  |  |  |  |

**Key:**

SOC = standard-of-care

RT = radiotherapy to the prostate

WHO = World Health Organization

PSA = prostate specific antigen

ADT = androgen deprivation therapy

IQR = interquartile range

Table B: Eligibility status following participant audit

| **Eligibility categorisation** | **SOC**  **(n=1029)** | **SOC+RT**  **(n=1032)** |
| --- | --- | --- |
| Eligible | 1011 (98%) | 1014 (98%) |
| Ineligible | 14 (1%) | 12 (1%) |
| Potentially ineligible | 4 (<1%) | 6 (1%) |

**Key:**

SOC = standard-of-care

RT = radiotherapy to the prostate

Table C: Sensitivity analyses on overall survival based on explicit eligibility

| **Analysis** | **Patient group** | **Deaths recorded** | | **Adjusted Hazard Ratio** |
| --- | --- | --- | --- | --- |
|  |  | **SOC** | **SOC+RT** |  |
| **All patients** | ITT (n=2061) | 609/1029 (59%) | 574/1032 (56%) | 0.90 (0.81 to 1.01) |
|  | Ineligible excluded (n=2025) | 599/1011 (59%) | 567/1014 (56%) | 0.91 (0.81 to 1.02) |
|  |  |  |  |  |
| **Lower metastatic burden** | ITT (n=819) | 202/409 (49%) | 156/410 (38%) | 0.64 (0.52 to 0.79) |
|  | Ineligible excluded (n=802) | 199/402 (50%) | 152/400 (38%) | 0.64 (0.52 to 0.79) |
|  |  |  |  |  |
| **Higher metastatic burden** | ITT (n=1120) | 375/567 (66%) | 386/553 (70%) | 1.11 (0.96 to 1.28) |
|  | Ineligible excluded (n=1104) | 368/557 (66%) | 383/547 (70%) | 1.11 (0.96 to 1.29) |
|  |  |  |  |  |

**Key:**

SOC = standard-of-care

RT = radiotherapy to the prostate

ITT = intention-to-treat

Table D: First Local Intervention event reported (patients with event reported)

| **Type of event** | **SOC**  **(n=556)** | **SOC+RT**  **(n=530)** |
| --- | --- | --- |
| Urinary catheter | 57 (10%) | 56 (11%) |
| TURP | 33 (6%) | 29 (5%) |
| Ureteric stent | 21 (4%) | 8 (2%) |
| Nephrostomy | 7 (1%) | 3 (1%) |
| Colostomy | 3 (1%) | 3 (1%) |
| Surgery for bowel obstruction | 0 (0%) | 2 (<1%) |
| PCa death | 435 (78%) | 429 (81%) |

**Key:**

SOC = standard-of-care

RT = radiotherapy to the prostate

TURP = transurethral resection of the prostate

PCa = prostate cancer

Table E: Summary of Analyses of Time to Local Event Outcomes

| **Outcome measure** | **Patient group** | **Analysis** | | **Adjusted Hazard Ratio*** |
| --- | --- | --- | --- | --- |
|  |  |  |  |  |
| **Symptomatic local event-free survival** | Overall | Cause-specific (including PCa death)^^^ | 1.00 (0.90 to 1.13) | |
|  |  | Cause-specific (excluding PCa death)^^^ | 1.04 (0.86 to 1.26) | |
|  |  | Competing-risks^+^ | 1.05 (0.86 to 1.27) | |
|  | Low metastatic burden | Cause-specific (including PCa death)^^^ | 0.72 (0.59 to 0.88) | |
|  |  | Cause-specific (excluding PCa death)^^^ | 0.79 (0.60 to 1.05) | |
|  |  | Competing-risks^+^ | 0.83 (0.62 to 1.10) | |
|  | High metastatic burden | Cause-specific (including PCa death)^^^ | 1.23 (1.06 to 1.42) | |
|  |  | Cause-specific (excluding PCa death)^^^ | 1.24 (0.94 to 1.64) | |
|  |  | Competing-risks^+^ | 1.21 (0.92 to 1.59) | |
| **Local intervention-free survival** | Overall | Cause-specific (including PCa death)^^^ | 0.94 (0.83 to 1.06) | |
|  |  | Cause-specific (excluding PCa death)^^^ | 0.83 (0.63 to 1.08) | |
|  |  | Competing-risks^+^ | 0.83 (0.64 to 1.08) | |
|  | Low metastatic burden | Cause-specific (including PCa death)^^^ | 0.62 (0.49 to 0.77) | |
|  |  | Cause-specific (excluding PCa death)^^^ | 0.61 (0.41 to 0.90) | |
|  |  | Competing-risks^+^ | 0.64 (0.43 to 0.94) | |
|  | High metastatic burden | Cause-specific (including PCa death)^^^ | 1.18 (1.01 to 1.37) | |
|  |  | Cause-specific (excluding PCa death)^^^ | 1.03 (0.70 to 1.51) | |
|  |  | Competing-risks^+^ | 0.99 (0.68 to 1.45) | |
|  |  |  |  | |

* Subdistribution hazard ratio for competing-risks models.

^ Cox model, adjusting for age, nodal involvement, WHO performance status, regular aspirin or NSAID use and planned SOC docetaxel at randomisation, stratified by randomisation time period.

+ Fine and Gray model with outcome excluding PCa death and death from any cause as competing risk.

Table F: Grade 3-5 late RT toxicities reported over entire time on trial (RTOG)

| **Toxicity Category** | **SOC**  **(n=977)** | **SOC+RT**  **(n=990)** |
| --- | --- | --- |
| **Urinary** | **4 (<1%)** | **20 (2%)** |
| Haematuria | 1 (<1%) | 8 (1%) |
| Urethral stricture | 2 (<1%) | 7 (1%) |
| Cystitis | 1 (<1%) | 7 (1%) |
| **Bowel** | **3 (<1%)** | **26 (3%)** |
| Proctitis | 1 (<1%) | 14 (1%) |
| Diarrhoea | 1 (<1%) | 12 (1%) |
| Rectal-anal stricture | 0 (0%) | 0 (0%) |
| Rectal ulcer | 0 (0%) | 1 (<1%) |
| Bowel obstruction | 1 (<1%) | 2 (<1%) |

**Note:** Treatment arms correspond to safety population; patients with ≥1 Follow-Up CRF returned.

**Key:**

SOC = standard-of-care

RT = radiotherapy to the prostate

RTOG = Radiation Therapy Oncology Group

Table G: Grade 3-5 late RT toxicities reported at 2 years (RTOG)

| **Toxicity Category** | **SOC**  **(n=375)** | **SOC+RT**  **(n=610)** |
| --- | --- | --- |
| **Urinary** | **0 (0%)** | **3 (<1%)** |
| Haematuria | 0 (0%) | 1 (<1%) |
| Urethral stricture | 0 (0%) | 2 (<1%) |
| Cystitis | 0 (0%) | 0 (0%) |
| **Bowel** | **0 (0%)** | **6 (1%)** |
| Proctitis | 0 (0%) | 3 (<1%) |
| Diarrhoea | 0 (0%) | 2 (<1%) |
| Rectal-anal stricture | 0 (0%) | 0 (0%) |
| Rectal ulcer | 0 (0%) | 1 (<1%) |
| Bowel obstruction | 0 (0%) | 0 (0%) |

**Note:** Treatment arms correspond to safety population; patients with ≥1 Follow-Up CRF returned and no reported progression at 2 years.

**Key:**

SOC = standard-of-care

RT = radiotherapy to the prostate

RTOG = Radiation Therapy Oncology Group

Table H: Grade 3-5 late RT toxicities reported at 4 years (RTOG)

| **Toxicity Category** | **SOC**  **(n=238)** | **SOC+RT**  **(n=467)** |
| --- | --- | --- |
| **Urinary** | **0 (0%)** | **0 (0%)** |
| Haematuria | 0 (0%) | 0 (0%) |
| Urethral stricture | 0 (0%) | 0 (0%) |
| Cystitis | 0 (0%) | 0 (0%) |
| **Bowel** | **1 (<1%)** | **2 (<1%)** |
| Proctitis | 0 (0%) | 0 (0%) |
| Diarrhoea | 1 (<1%) | 1 (<1%) |
| Rectal-anal stricture | 0 (0%) | 0 (0%) |
| Rectal ulcer | 0 (0%) | 0 (0%) |
| Bowel obstruction | 0 (0%) | 1 (<1%) |

**Note:** Treatment arms correspond to safety population; patients with ≥1 Follow-Up CRF returned and no reported progression at 4 years.

**Key:**

SOC = standard-of-care

RT = radiotherapy to the prostate

RTOG = Radiation Therapy Oncology Group

Table I: Grade 3-5 adverse events reported over entire time on trial, overall and for selected body systems (CTCAE)

| **Body system** | **SOC**  **(n=1052)** | **SOC+RT**  **(n=992)** |
| --- | --- | --- |
| **Overall** | **458 (44%)** | **451 (45%)** |
| Blood & bone marrow | 56 (5%) | 49 (5%) |
| Cardiovascular | 46 (4%) | 56 (6%) |
| Endocrine | 160 (15%) | 155 (16%) |
| Gastrointestinal | 47 (4%) | 52 (5%) |
| General | 57 (5%) | 43 (4%) |
| Lab abnormalities | 49 (5%) | 48 (5%) |
| Musculoskeletal & connective tissue | 112 (11%) | 104 (10%) |
| Renal | 50 (5%) | 52 (5%) |

**Note:** Treatment arms correspond to safety population; patients with ≥1 Follow-Up/SAE CRF returned.

**Key:**

SOC = standard-of-care

RT = radiotherapy to the prostate

RTOG = Radiation Therapy Oncology Group

**Table J: Grade 3-5 adverse events reported at 2 years, overall and for selected body systems (CTCAE)**

| **Body system** | **SOC**  **(n=320)** | **SOC+RT**  **(n=395)** |
| --- | --- | --- |
| **Overall** | **52 (16%)** | **54 (14%)** |
| Blood & bone marrow | 0 (0%) | 0 (0%) |
| Cardiovascular | 9 (3%) | 5 (1%) |
| Endocrine | 32 (10%) | 35 (9%) |
| Gastrointestinal | 1 (<1%) | 3 (1%) |
| General | 3 (1%) | 3 (1%) |
| Lab abnormalities | 0 (0%) | 0 (0%) |
| Musculoskeletal & connective tissue | 4 (1%) | 4 (1%) |
| Renal | 3 (1%) | 2 (1%) |

**Note:** Treatment arms correspond to safety population; patients with ≥1 Follow-Up/SAE CRF returned and no reported progression at 2 years.

**Key:**

SOC = standard-of-care

RT = radiotherapy to the prostate

CTCAE = Common Terminology Criteria for Adverse Events

**Table K: Grade 3-5 adverse events reported at 4 years, overall and for selected body systems (CTCAE)**

| **Body system** | **SOC**  **(n=133)** | **SOC+RT**  **(n=225)** |
| --- | --- | --- |
| **Overall** | **12 (9%)** | **29 (13%)** |
| Blood & bone marrow | 0 (0%) | 0 (0%) |
| Cardiovascular | 0 (0%) | 5 (2%) |
| Endocrine | 10 (8%) | 20 (9%) |
| Gastrointestinal | 0 (0%) | 1 (<1%) |
| General | 2 (2%) | 1 (<1%) |
| Lab abnormalities | 0 (0%) | 0 (0%) |
| Musculoskeletal & connective tissue | 2 (2%) | 1 (<1%) |
| Renal | 0 (0%) | 1 (<1%) |

**Note:** Treatment arms correspond to safety population; patients with ≥1 Follow-Up/SAE CRF returned and no reported progression at 4 years.

**Key:**

SOC = standard-of-care

RT = radiotherapy to the prostate

CTCAE = Common Terminology Criteria for Adverse Events
