# Supplementary material for: Radiotherapy to the prostate for men with metastatic prostate cancer in the UK and Switzerland: Long-term results from the STAMPEDE randomised controlled trial
Source: PLoS Med. 2022 Jun 7;19(6):e1003998. doi: 10.1371/journal.pmed.1003998 (PMC9173627; doi:10.1371/journal.pmed.1003998)
Supplement: S2 Text — (PDF) [file pmed.1003998.s013.pdf]

DocuSign Envelope ID: 67C184DE-98FB-4FA6-BD6F-B91732928013

(Logos removed to comply with journal requirements)

(Logos removed to comply with journal requirements)

(Logos removed to comply with journal requirements)

STAMPEDE

Systemic Therapy in Advancing or Metastatic Prostate Cancer: Evaluation of Drug Efficacy
A multi-arm multi-stage randomised controlled trial

MRC PR08

ISRCTN number:	ISRCTN78818544
EUDRACT number:	2004-000193-31
CTA number:	00316/0026/001-0001
NCT number:	NCT00268476

STATISTICAL ANALYSIS PLAN
-- “M1|RT comparison” long-term follow-up ---

MRC Clinical Trials Unit at UCL

Tel: +44 (0)20 7670 4798
Fax: +44 (0)20 7670 4818
Email: mrcctu.stampede@ucl.ac.uk

Version date: Version 1.0; 01-Mar-2021

Contacts
Matthew Sydes
Adrian Cook
Chris Brawley

This template and all preceding versions will be stored in the Statistical Analysis Master File for this trial held in S:\MRCCTU_Stampede_Stats\SAP

DocuSign Envelope ID: 67C184DE-98FB-4FA6-BD6F-B91732928013

CONTENTS

1 ABBREVIATIONS 4

2 INTRODUCTION 6
2.1 COMPARISONS 6
2.2 METHOD OF RANDOMISATION FOR STAMPEDE TRIAL 7

3 OUTCOME MEASURES 9

4 ANALYSIS PRINCIPLES 13
4.1 OUTLINE ANALYSIS PLAN 13
4.2 TIME PERIODS 13
4.3 PERIOD OF DATA COLLECTION 15
4.4 MAIN ANALYSIS 15
4.5 LONG-TERM ANALYSIS 15
4.6 STATISTICAL METHODS 15
4.6.1 Time-to-event analyses 15
4.6.2 Proportional and non-proportional hazards 16
4.6.3 Competing Risks 17
4.7 PATIENTS WITH NO DATA 17
4.8 MATURITY 17
4.9 DEATHS AND CAUSE OF DEATH 17
4.9.1 Data linkage for death 18
4.10 POPULATIONS 18
4.11 SAFETY ANALYSES 19
4.12 MISSING DATA 21
4.13 PRE-SPECIFIED SUBGROUP EFFECTS 21
4.13.1 Metastatic volume 21
4.13.2 Radiotherapy Schedule 22
4.13.3 Stratification Factors 23
4.13.4 Other Subgroup Analyses 23
4.14 COMPARATIVE ANALYSIS OF QUALITY-OF-LIFE DATA 23
4.14.1 Data sources 23
4.14.2 data completion schedule 23
4.14.3 data verification 24
4.14.4 coverage of analysis 24
4.14.5 populations 25
4.14.6 missing data 25
4.14.7 main outcome measure for Quality-of-life analysis 25
4.14.8 secondary outcome measures for Quality-of-life analysis 26
4.14.9 Minimally important difference 30

5 ANALYSIS DETAILS 31
5.1 PRE-PLANNED DATA CHECKS 31
5.2 PLANNED ANALYSES 31
5.2.1 Baseline Characteristics 31
5.2.2 Standard-of-care Treatment 32
5.2.3 Trial Treatment 33
5.2.4 Trial Events 33
5.2.5 Toxicity/Safety 35
5.2.6 Data Returns 36

DocuSign Envelope ID: 67C184DE-98FB-4FA6-BD6F-B91732928013

5.2.7 Quality-of-life analyses 36
5.3 EXPLORATORY ANALYSES 37

6 SIGNATURES OF APPROVAL 38

1 ABBREVIATIONS

Abbreviation	Expansion
Abi	Abiraterone
ADT	Androgen-deprivation therapy
AMP	Adenosine monophosphate
AS	Activity Stage
BMD	Bone mineral density
CCI	Comparison Chief Investigator
Cel	Celecoxib
CHF	Congestive heart failure
CI	Chief Investigator
CI	Confidence interval
CRF	Case Report Form
CTCAE	Common Toxicity Criteria for Adverse Events
CTU	Clinical Trials Unit
CV	Cerebrovascular
DAB	Dual Androgen Blockade
DMP	Data Management Plan
Doc	Docetaxel
DAB	Dual Androgen Blockade (previously Maximum Androgen Blockade [MAB])
Enza	Enzalutamide
ES	Efficacy Stage
FFS	Failure-free survival
FPM	Flexible parametric models
HE	Health Economics
HEAP	Health Economics Analysis Plan
HR	Hazard ratio
HT	Hormone therapy
IDMC	Independent Data Monitoring Committee
ITT	Intention-to-treat
KM	Kaplan-Meier
LHRH	Luteinising hormone-releasing hormone
LOB	Lack-of-benefit
M0	Non-metastatic
M1	Metastatic
MACE	Major adverse cardiac event
MAMS	Multi-arm multi-stage
MCAR	Missing completely at random
MI	Myocardial infarction
MPFS	Metastatic progression-free survival
MRC	Medical Research Council
N+	Lymph node-positive
N0	Lymph node-negative
NX	Lymph node stage unknown
NSAID	Non-steroidal anti-inflammatory drug
ONS	Office of National Statistics
OS	Overall survival
PCa	Prostate cancer
PH	Proportional hazards
PHE	Public Health England
PSA	Prostate specific antigen
q6wk	Every 6 weeks
q12wk	Every 12 weeks

DocuSign Envelope ID: 67C184DE-98FB-4FA6-BD6F-B91732928013

Abbreviation	Expansion
**q6m**	Every 6 months
**q12m**	Every 12 months
**QL**	Quality of Life
**RMST**	Restricted mean survival time
**rPFS**	Radiological progression-free survival
**RT**	Radiotherapy
**RTOG**	Radiation Therapy Oncology Group
**SAE**	Serious adverse event
**SAP**	Statistical Analysis Plan
**SOC**	Standard-of-care
**SOP**	Standard Operating Procedures
**TBD**	To be determined
**TMG**	Trial Management Group
**TSC**	Trial Steering Committee
**WHO PS**	WHO Performance Status
**ZA**	Zoledronic acid

2 INTRODUCTION

This statistical analysis plan (SAP) describes only the long-term follow-up analysis of the "M1|RT comparison". When the sample size was increased during recruitment, a further analysis on overall survival was planned for approximately 18 months after overall survival would be examined in the patient group overall. This was partly to enable an analysis within one or both patient subgroups defined by planned RT schedule (daily, 55Gy/20F, or weekly, 36Gy/6F) when ~199 control arm deaths were observed, provided there was evidence of a treatment effect on FFS within the relevant subgroup. At the time of the primary analysis in June-2018, there were not enough control arm deaths to perform this analysis in both RT-scheduled-based subgroups.

Full details of the background to the trial and the primary analysis of the "M1|RT comparison" can be found in the STAMPEDE "M1|RT comparison" SAP v3.1, dated 05-Jun-2018.

2.1 COMPARISONS

A research comparison is defined by those patients allocated to the research arm, along with the corresponding contemporaneously randomised, eligible control arm patients. See **Table 1** for the definition of each research comparison within STAMPEDE to date.

Table 1: STAMPEDE Research Comparisons

Comparison name	Included Arms	Eligible patients	Accrual	
			Start Date	End Date
"Zoledronic acid comparison"	A, B	All patients	05-Oct-2005	31-Mar-2013
"Docetaxel comparison"	A, C	All patients	05-Oct-2005	31-Mar-2013
"Celecoxib comparison"	A, D	All patients	05-Oct-2005	06-Apr-2011
"Zoledronic acid + docetaxel comparison"	A, E	All patients	05-Oct-2005	31-Mar-2013
"Zoledronic acid + celecoxib comparison"	A, F	All patients	05-Oct-2005	06-Apr-2011
"Abiraterone comparison"	A, G	All patients	15-Nov-2011	17-Jan-2014*
"M1|RT comparison"	A, H	Newly-diagnosed M1 pts<br>No contraindication to RT	22-Jan-2013	02-Sep-2016
"Enzalutamide + abiraterone comparison"	A, J	All patients	29-Jul-2014	31-Mar-2016
"Metformin comparison"	A, K	Non-diabetic pts<br>No contraindication to metformin	05-Sep-2016	TBD
"tE2 comparison"	A, L	<8wk anti-androgen use<br>Maximum 4wk LHRH t'py<br>No bilateral orchidectomy	20-Jun-2017	TBD

***Note:** One patient was manually randomised to Arm G after the cut-off of 17-Jan-2014

D, F: The celecoxib-containing arms closed accrual early due to lack of sufficient activity following their Activity Stage 2 analysis.

B, C, E: The remaining original research arms closed to recruitment having reached an acceptable sample size (based on time to analysis projections).

G: The abiraterone arm closed to recruitment having reached its revised sample size target (1800 pts) ahead of schedule.

H: The M1|RT arm closed to recruitment having reached its revised target sample size ahead of schedule.

J: The enzalutamide + abiraterone arm closed to recruitment having reached its target sample size ahead of schedule.

DocuSign Envelope ID: 67C184DE-98FB-4FA6-BD6F-B91732928013

Figure 1: Activity-by-time graph showing patients contributing to this comparison

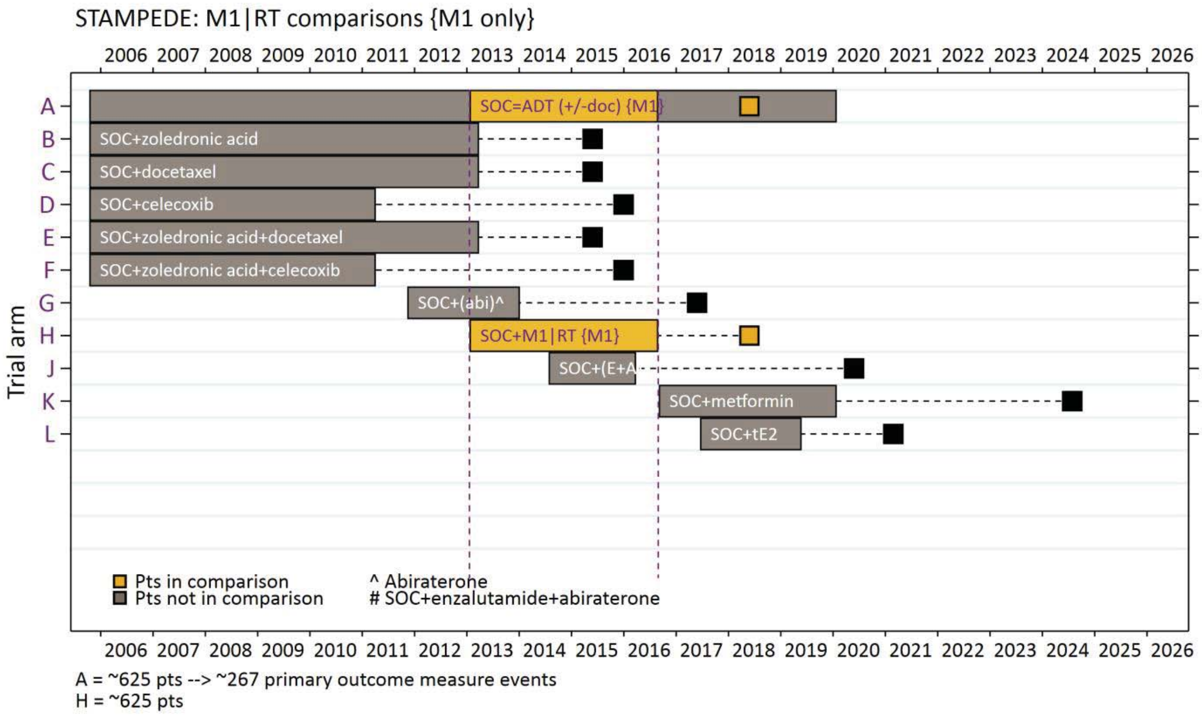

2.2 METHOD OF RANDOMISATION FOR STAMPEDE TRIAL

Patients are randomised to STAMPEDE centrally using a computerised algorithm developed and maintained by the CTU. Randomisation is performed using the method of minimisation over a number of clinically important stratification factors with an additional random element. These factors are:

• Randomising centre	each centre
• Metastases	M0 vs M1
• Nodal involvement	N0 vs NX vs N+
• Age at randomisation	up to 69yrs vs 70yrs and over
• WHO performance status	PS=0 vs PS=1-2
• Method of ADT[1]	Orchidectomy vs LHRH agonist vs LHRH antagonist vs Dual Androgen Blockade (DAB)
• Regular aspirin or NSAID use at baseline	yes vs no
• Radiotherapy planned[2]	yes vs no
• Docetaxel planned[3]	yes vs no

[1] Method of ADT options have changed over time, from LHRH vs orchidectomy, to then include bicalutamide, then specify LHRH agonist or antagonist and more recently exclude bicalutamide but include DAB; see Stratification_Factors_OverTime.docx

[2] "Radiotherapy planned" was added as a stratification factor at the start of recruitment to Efficacy Stage I for the "original comparisons" (Mar-2008)

[3] Docetaxel planned was added as a stratification factor from 17-Dec-2015 following publication of the "original comparisons" results indicating docetaxel improved overall survival. In Nov-2018, after the "M1|RT comparison"

DocuSign Envelope ID: 67C184DE-98FB-4FA6-BD6F-B91732928013

When implementing the additional random element of the randomisation, an 80% probability of allocation will be split between the (one or more) arms the patient is eligible for with the lowest strata totals (i.e. 80% probability of being allocated to one of the minimising arms); and the remaining 20% probability of allocation will be split between the remaining (one or more) eligible arms. The balance is maintained separately for each of the combinations of arms between which patients can be randomised. This method should provide simplicity of reporting and implementation.

had closed for recruitment this stratification factor was updated to 'Standard-of-care docetaxel or abiraterone planned', with the potential options being docetaxel vs abiraterone vs neither.

3 OUTCOME MEASURES

The "M1|RT comparison" seeks to assess for improved overall survival (all-cause mortality) and therefore this forms the definitive primary outcome measure for the comparison.

The fully specified outcome measures for this comparison in the protocol are listed in the **Table 2**, below. Definitions are provided in **Table 3**.

Table 2: "M1|RT comparison" outcome measures by comparison stage

COMPARISON STAGE	PRIMARY OUTCOME MEASURE	SECONDARY OUTCOME MEASURES[T]
**Pilot phase(s)**	Safety	Feasibility
**Activity Stages**	Failure-free survival (FFS)[†]	Overall survival (OS)[x]<br>Biochemical Failure<br>Progression-free survival<br>Disease-specific survival<br>Non-PCa death<br>Lymph node progression<br>Distant metastases (Metastatic Progression-Free Survival)<br>Toxicity<br>Symptomatic skeletal events<br>Therapy for progression
**Efficacy Stage**	Overall survival[x]	Failure-free survival[†]<br>Other 2^0 OMs as for Activity Stages plus:<br>Symptomatic local event (SLE)-free survival<br>Quality of life<br>Cost effectiveness
**Long-term follow-up**	Overall survival[x]	Symptomatic local event-free survival<br>Local intervention-free survival<br>Long-term toxicity, focusing on urinary, bowel and sexual function symptoms, as reported on Follow-up CRF using RTOG system and using standard CTCAE system for (serious) adverse events<br>Quality-of-life

[x] Cause of death with a view to cause-specific survival from PCa (with death from other causes as a competing risk) is considered under this.

DocuSign Envelope ID: 67C184DE-98FB-4FA6-BD6F-B91732928013

Table 3: Definitions of certain terms (ordered by primary outcome, secondary outcome & other terms)

Term	Definition
Overall survival (OS)	Time from randomisation until death from any cause. For surviving patients, censor date is used; if ONS data is available use censor date 2, otherwise use censor date 1 (see below).
Failure-free Survival (FFS)	Time from randomisation until first of the following events:<br>• Biochemical failure (as defined in protocol)<br>• Local progression<br>• Lymph node progression<br>• Distant metastases<br>• Skeletal Related Event (where confirmed disease progression)<br>• Death from prostate cancer<br>For patients who have not had an event, censor date is used (see below).<br>If a suspicious event is reported for any of local progression, lymph node progression, distant metastases progression this will be counted as a FFS event.
Progression-free Survival (PFS)	Time from randomisation until first of:<br>• Local progression<br>• Lymph node progression<br>• Distant metastases<br>• Skeletal Related Event (where confirmed disease progression)<br>• Death from prostate cancer<br>For patients who have not had an event, censor date is used (see below).<br>If a suspicious event is reported for any of local progression, lymph node progression, distant metastases progression this will be counted as a PFS event.
Metastatic Progression-Free Survival (MPFS)	Time from randomisation until first of:<br>• Distant metastases<br>• Skeletal Related Event (where confirmed disease progression)<br>• Death from prostate cancer<br>For patients who have not had an event, censor date is used (see below).<br>If a suspicious event is reported for distant metastases progression this will be counted as a MPFS event.
Metastasis-Free Survival (MFS)	Time from randomisation until first of:<br>• Radiologically-confirmed distant metastases<br>• Death from any cause
Skeletal Related Event	• Bone pain requiring radiotherapy and/or surgery<br>• Pathological fracture<br>• Metastatic spinal cord compression
Disease-specific survival	Time from randomisation until death from prostate cancer (see below).<br>For patients who have not had an event, censor date is used; if ONS data is available use censor date 2, otherwise use censor date 1 (see below).

DocuSign Envelope ID: 67C184DE-98FB-4FA6-BD6F-B91732928013

TERM	DEFINITION
Death from prostate cancer	In the "M1|RT comparison", death from prostate cancer is determined by the primary cause of death reported by the site on the Death CRF. Deaths with prostate cancer reported as the primary cause are treated as being due to prostate cancer for the relevant outcomes; deaths with any other primary cause of death reported on the CRF are treated as not due to prostate cancer. To account for issues with the Death CRF not being completed correctly, the primary cause of death was amended to 'prostate cancer' in the following circumstances:<br>• Primary cause of death not categorised as prostate cancer but "prostate cancer" specified in accompanying freetext field<br>• "Carcinomatosis" specified as primary cause of death, with prostate cancer given as secondary cause<br>• If death was reported as being treatment-related on Death CRF<br>• If neutropenic sepsis was reported as the primary, secondary or tertiary cause of death<br>• If (broncho)pneumonia reported as primary cause of death, prostate cancer the secondary or tertiary cause and patient had progressed by the time of death
Symptomatic Local Event-Free Survival (SLEFS)	Time from randomisation until first of:<br>• TURP<br>• Ureteric stent<br>• Surgery for bowel obstruction<br>• Urinary catheter<br>• Nephrostomy<br>• Colostomy<br>• Acute kidney injury<br>• Urinary tract infection<br>• Urinary tract obstruction<br>• Urostomy / ileal conduit[4]<br>• Death from prostate cancer
Local Intervention-Free Survival (LIFS)	Time from randomisation until first of:<br>• TURP<br>• Ureteric stent<br>• Surgery for bowel obstruction<br>• Urinary catheter<br>• Nephrostomy<br>• Colostomy<br>• Death from prostate cancer
Censor date 1	Date taken from the latest of the relevant variables defined below:<br>• Date of randomisation (Form 1)<br>• BMD assessment date (scan, blood sample, urine sample)<br>• Date of treatment cycle (as taken from the bisphosphonate, docetaxel; Forms 4, 5, 6)<br>• Date bloods taken (as taken from the bisphosphonate Forms 4, 5)<br>• Date of last SOC docetaxel cycle (Form 21)<br>• Date of any treatment action (Forms 7, 7B, 7C, 7D)<br>• Date of any tE2 treatment action for Arm L patients (Form 25)<br>• Date of tests recorded on hormone results log for Arm L patients (Form 24)<br>• Dates reported on the Follow-up CRF (including date of PSA tests, date of any surgical interventions, date of any SRE, date of any metabolic or cardiovascular event; Forms 7, 7A)

[4] Information on surgical procedures and toxicities that comprise the Symptomatic Local Event outcome measure is collected routinely on Follow-up CRF or as prompted sub-category on Toxicity CRF (urinary tract obstruction was first added to list of toxicities collected when separate Toxicity CRF was introduced in Sep-2016), except for urostomy / ileal conduit. Analysis of this outcome measure will exclude urostomy / ileal conduit, but in future information on this procedure may be obtained from PHE, in which case the analysis will be updated to include this.

DocuSign Envelope ID: 67C184DE-98FB-4FA6-BD6F-B91732928013

TERM	DEFINITION
	• Date of any reported progression event (Form 8)<br>• Date additional treatment started or stopped (Forms 8, 8A)<br>• Date of first/last RT fraction (Form 9A)<br>• Date of late RT toxicity assessment (Form 10)<br>• Date HT/research treatment ended (Form 11)<br>• SAE date (onset, resolved, recent HT or trial treatment administration, start/end date of other treatment, test date) (Form 14)<br>• Date of palliative RT fraction (Form 19)<br>• Date blood or saliva sample obtained as reported on the pathology form (Form 18)<br>• Date of co-enrolment to another trial (Form 15)<br>• Date trial participation ended (Form 20)<br>• Date last known alive (Form 7 from Version 13.0)<br>• Date of death (Censoring date only for outcomes other than overall survival and disease-specific survival; Form 12)<br>**Notes**:<br>• Dates from the QoL forms are no longer used as a censor date as these are completed by the patient and cannot be queried for errors.<br>• Dates of form completion are no longer used as the CRF may have been completed retrospectively.<br>• Any date pre-randomisation is ignored within the calculation.<br>• Unusual dates which have not yet been resolved or dates after the date of the corresponding data freeze will be ignored for the purposes of calculating this censor date.
Censor date 2	• For patients with successful flagging with ONS (or equivalent) a censoring date will be set at 4 or 8 weeks before the ONS data transfer, as ONS advise, if this is earlier than 30-Nov-2020, the date on which follow-up for the comparison was stopped, or on 30-Nov-2020 otherwise.
Stratification factors[5]	• Randomising centre<br>• Metastases at randomisation (Not "M1|RT comparison")<br>• Nodal involvement<br>• Age at randomisation<br>• WHO performance status<br>• Method of hormones<br>• Regular aspirin or NSAID<br>• Radiotherapy planned (from Mar-2008; Not "M1|RT comparison")<br>• SOC Docetaxel or abiraterone or neither planned (docetaxel from 17-Dec-2015; abiraterone added 26-Nov-2018)
Frozen Dataset	A copy of the data used for an analysis and to which no *new* CRFs are added. Data queries on the frozen copy should still be resolved, wherever possible, up to the point where the analysis is actually performed. Any changes will be hard-coded into the analysis files and highlighted and annotated as well as updating the live database (for future analyses).

[5] All stratification factors that have been used to randomise patients to STAMPEDE. Note that this list has grown over time; details of the stratification factors over time are outlined in the following document: "S:\MRCCTU_Stampede_Stats\SAP\Stratification_Factors_OverTime - v1.0.doc"

DocuSign Envelope ID: 67C184DE-98FB-4FA6-BD6F-B91732928013

4 ANALYSIS PRINCIPLES

4.1 OUTLINE ANALYSIS PLAN

All efficacy analyses will be performed on an intention-to-treat basis. For the analysis of this comparison, the group defined by patients allocated to research arm H (SOC+prostate RT) will be compared against the corresponding control arm A group.

The process for analyses of FFS and OS will follow that proposed by Royston and Parmar (*Statist. Med.* 2001, 30 2409-2421).

A stratified log-rank test for a difference in survival by allocated treatment (stratifying across the factors used in randomisation (except for centre and choice of HT) and time period at randomisation [see below]) will be performed.

Kaplan-Meier (KM) plots will be used to visually assess a survival difference between the two groups, formatted following the KMunicate structure (Morris 2019 *BMJ Open*). Evidence against the proportional hazards assumption will be tested using the Grambsch-Therneau test after fitting an adjusted Cox model, and a suitable method of summarising a difference between the treatment groups determined accordingly based on criteria outlined below.

All statistical tests for this analysis will be implemented using a 2-sided p-value of 0.05, unless otherwise specified. There will be no formal adjustment of p-values because of the interim analyses performed, as this has been pre-considered in the design. A note can be made of the familywise error rate (FWER) associated with a comparison in its context.

4.2 TIME PERIODS

Analyses will be stratified by or adjusted for the time periods during which patients were randomised between upfront prostate RT and standard-of-care. Time periods are defined by trial arms opening or closing, a change to the standard-of-care, or another fundamental aspect which may affect the patient population being randomised. Recruitment to the "M1|RT comparison" occurred across periods 4, 5, 6, 7, 8 and 9. Due to technical limitations with the statistical software used at the time of the primary analysis in 2018 and the long-term follow-up analysis in 2021, periods 4 and 5 will be combined for the purpose of fitting stratified Cox models. Period 4 contains the fewest patients and covers the shortest length of time.

Figure 1: Arms active over time (time strata)

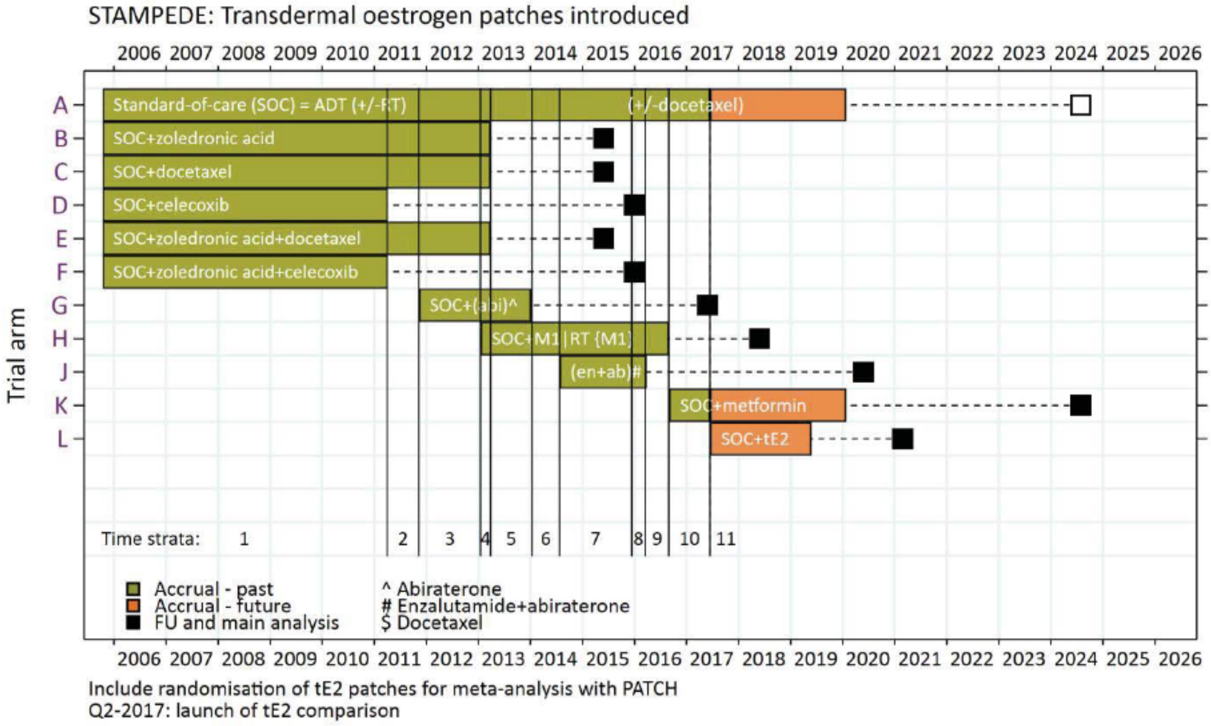

Table 4: Time periods within STAMPEDE (relevant accrual time periods are highlighted in yellow)

Time Period	Definition	Accrual Start Date	Accrual End Date	Co-recruiting research arms
1	From the start of the trial up to the stopping of the celecoxib-containing research Arms D & F	05-Oct-2005	06-Apr-2011	B C D E F
2	Post-closure of Arms D & F up to the opening of the abiraterone research Arm G	06-Apr-2011	14-Nov-2011	B C E
3	Post-opening of Arm G up to the opening of the M1 radiotherapy research Arm H	15-Nov-2011	21-Jan-2013	B C E G
4	Post-opening of Arm H up to the closure of the remaining original research Arms B, C & E	22-Jan-2013	31-Mar-2013	B C E G H
5	Post-closure of Arms B, C & E up to the closure of abiraterone research Arm G	01-Apr-2013	17-Jan-2014*	G H
6	Post-closure of Arm G up to the opening of the enzalutamide+abiraterone research Arm J	18-Jan-2014	28-Jul-2014	H
7	Post-opening of Arm J up to the update in SOC to permit planned use of docetaxel as first line treatment	29-Jul-2014	16-Dec-2015	H J
8	Post-update of SOC up to the closure of enzalutamide+abiraterone research Arm J	17-Dec-2015	31-Mar-2016	H J
9	Post closure of Arm J up to the close of M1|RT research Arm H and opening of metformin research Arm K	01-Apr-2016	02-Sep-2016	H
10	Post-opening of Arm K to the opening of transdermal oestradiol research arm L	05-Sep-2016	19-Jun-2017	K
11	Post-opening of Arm L to the update in SOC permitting planned use of abiraterone or docetaxel as first-line treatment	20-Jun-2017	25-Nov-2018	K L
12	Post-update of SOC onwards to pausing of recruitment to "metformin comparison"	26-Nov-2018	25-Oct-2019	KL
13	Post pausing of recruitment to arm K, to suspension of all recruitment due to covid-19 pandemic	26-Oct-2019	31-Mar-2020	L

***Note:** One patient was manually randomised to Arm G after the cut-off of 17-Jan-2014

DocuSign Envelope ID: 67C184DE-98FB-4FA6-BD6F-B91732928013

4.3 PERIOD OF DATA COLLECTION

The start date was 22-Jan-2013, the date the "M1|RT comparison" was opened to recruitment. The cut-off date for sites to collect follow-up data for arm H patients and arm A patients who are controls for the "M1|RT comparison" only was 30-Nov-2020. Therefore the long-term analysis of the comparison will be based on events from 22-Jan-2013 and 30-Nov-2020, inclusive. No further follow-up information was collected beyond this date for these patients, though CRF entry and querying of information relating to actions prior to the cut-off date was continued in order to maximise the completeness of data for analysis. For arm A patients who are also controls in the "abiraterone comparison" or "enzalutamide+abiraterone comparison" data collection will continue after 30-Nov-2020, but the data used for the long-term analysis of the "M1|RT comparison" will exclude actions / events / assessments that occurred after this point. The date of database lock for the long-term follow-up analysis of the comparison is 17-Mar-2021. (Note that, if available, data from ONS may be frozen later than this point but would be wound back to the same cut-off date for analysis.)

4.4 MAIN ANALYSIS

The primary efficacy analysis of the "M1|RT comparison" was triggered by a pre-specified number of primary outcome events reported for control arm patients.

4.5 LONG-TERM ANALYSIS

The long-term analysis was planned to take place approximately 18 months after the main analysis, at which point a meaningful amount of extra information was expected to be available for analysis of overall survival within each subgroup defined by planned RT schedule, and to enable a fuller understanding of the long-term relationship between the treatment groups. This would also allow greater understanding by metastatic volume, particularly low volume metastatic patients, and of radiotherapy schedule within this group. This analysis will follow the principles of the long-term analyses of the "docetaxel comparison" and "abiraterone comparison", which were presented in 2019 and 2020 respectively.

4.6 STATISTICAL METHODS

4.6.1 TIME-TO-EVENT ANALYSES

Time-to-event data will first be analysed for evidence of a difference in survival by allocated treatment group using a log-rank test, stratified across all minimisation factors at randomisation (excluding randomising centre and type of long-term hormone therapy) and the relevant time periods for the "M1|RT comparison".

A visual depiction of survival over time will be presented using Kaplan-Meier plots. Time to most recent assessment will be used in all time-to-event analyses for patients that have not experienced the event in question (e.g. progression, death). Patients with no follow-up information will have a time of 0 days and therefore will not contribute to estimation of treatment effect for any outcome measure; those classified as experiencing "immediate" biochemical failure also effectively do not contribute to the estimate of treatment

DocuSign Envelope ID: 67C184DE-98FB-4FA6-BD6F-B91732928013

effect for failure-free survival. This means that any estimates of treatment effect are within the population of patients for whom follow-up information is available rather than the full ITT population, where at least one patient has time = 0. Given the trial team's efforts to ensure sites adhere to the schedule of assessments for patients set out in the protocol, there should be very little technical and no practical difference between these estimands.

For KM plots, all patients randomised to the comparison being analysed will be included. Those patients who have no reported event and contribute no information, such that they are censored at the date of randomisation, will be censored with a time of 0.001 days. The KMunicate approach to laying out risk tables will be used.

4.6.2 PROPORTIONAL AND NON-PROPORTIONAL HAZARDS

Evidence against the proportional hazards assumption will be quantified using the Grambsch-Therneau test after fitting a Cox proportional hazards model, adjusted for stratification factors and stratified by the relevant time periods. Application in Stata will be using the command `-estat phtest-` using log-transformed time, with evidence of non-PH from a global test being the main focus. The hazard ratio (HR) for treatment effect over time will be plotted, as estimated from a flexible parametric model with time-dependent treatment effect. As standard with an "omnibus approach", the model will utilise 5 degrees of freedom for the baseline distribution and 5 degrees of freedom for the time-dependent treatment effect. If the observed fit to the KM survival plots is unsatisfactory, these parameters and other aspects of the model may be modified to improve the fit. Details of any changes made will be highlighted.

A HR with 2-sided 95% confidence interval from the adjusted, time-stratified Cox model will be presented. This will be the main summary estimate of the treatment effect if there is no evidence of non-proportional hazards at the 5% significance level (i.e. $p<0.05$). The number of events observed and, to facilitate meta-analyses, the log-rank expected number of events and V will be presented.

Sensitivity analyses using alternative estimation of the treatment effect will also be presented e.g. log-rank hazard ratio. Flexible parametric models, modelling the difference between treatment groups, will be fitted to the time-to-event data with and without including time-dependent treatment effects.

If there is evidence of non-PH in the treatment effect, HRs are difficult to interpret and the restricted mean survival time[6] (RMST) (or "conditional expectation of time-to-event") difference constructed from the flexible parametric model with time-dependent treatment effect will be emphasised as the main estimate of treatment effect. This model will adjust for the stratification factors used at randomisation (except for centre and method of hormones) and relevant time strata, to determine the time-dependent treatment effect and then predict values needed for subsequent RMST analysis. Application in Stata will be using the command `–strmst–`. The time within which RMST will be computed, t*, will be determined by the observed timing of events in the control arm. This will be identified using the command `–maturity_rmst–` in Stata, developed within the CTU;

this is used to determine the maximum available follow-up time (if clinically meaningful) where there is most power for the analysis, or a suitable salient time point otherwise, for the comparison at the time of the analysis.

4.6.3 Competing Risks

Analysis of those outcomes where there are considered to be competing risks will be performed using a cause-specific survival analysis with an adjusted Cox model as well as a competing risks regression model using the Fine and Gray method, with a competing risk defined as any event the patient could likely have experienced that would preclude observation of the outcome of interest.

Outcome measures with competing risks are:

- Disease-specific survival: competing risk is death from other causes
- Non-PCa death: competing risk is death from PCa
- Lymph node progression: competing risk is distant metastatic progression or all-cause mortality
- Metastatic progression-free survival: competing risk is death from non-PCa causes
- Symptomatic local event: competing risk is death from non-PCa causes

4.7 PATIENTS WITH NO DATA

All models used to estimate treatment effect will include those individuals with no reported outcome event and contributing no censoring information such that they are censored at their date of randomisation (t=0). For the purpose of graphing only, these patients will be censored at t=0.001.

4.8 MATURITY

Median follow-up time will be calculated using a "reversed" Kaplan-Meier approach, taking censor date (if alive) to be an event and death as the time of censoring. The median follow-up time will be detailed by arm and within any pre-defined sub-groups of interest. In patients who were last known to be alive, time to last follow-up will be presented using standard summary statistics. Date last seen (if alive) is as defined within the censor date.

4.9 DEATHS AND CAUSE OF DEATH

For the "M1|RT comparison", all patients in the comparison have metastatic disease at randomisation and it is expected that the majority will die from prostate cancer (as evidenced in the patients not in this comparison but already reported). For the "M1|RT comparison", no central review of cause of death will be performed and the site classification will be accepted, with some corrections applied automatically to mitigate against incorrect completion of the Death CRF (see definition above). This reflects the greater certainty in ascertaining cause-of-death in this patient population.

DocuSign Envelope ID: 67C184DE-98FB-4FA6-BD6F-B91732928013

4.9.1 DATA LINKAGE FOR DEATH

Data from NHS Digital will be used, when and/or where available, to confirm deaths, detect unreported deaths and determine cause of death (if unknown and/or unreported). Where such external data sources are used for analyses of survival or disease-specific survival, patients who are not confirmed by this data source as having died are administratively censored close to the time of receipt of this external data; the exact time is determined in discussion with the provider of such data and should be reported explicitly. For example, for two previous CTU-led trials in prostate cancer, survival data were available from ONS and, in discussion with the ONS Chief Statistician in 2009, it was reasonable to assume that patients were still alive 8 weeks before data transfer if they were not reported as having died. This approach accepted that deaths referred to a coroner for review would have been inappropriately classified as censored rather than dead (with the time of censoring possibly later than the actual time of death). Therefore, any information received from sites subsequent to a data transfer from ONS (or equivalent) would be ignored.

In this analysis of the "M1|RT comparison" the main survival analyses will only use data collected within the trial, as per the primary analysis in 2018. A sensitivity analysis of Overall Survival will use external information on events and censoring for patients for whom data linkage with NHS Digital has been possible, and within-trial data for all other patients. Patients with linked data for whom an event has not been recorded will be administratively censored 4 weeks prior to the date of data transfer or on 30-Nov-2020 (the date that follow-up for the comparison stopped), whichever is earliest. (This is equivalent to Censor date 2 in Table 3.) Patients with within-trial data only will be censored at the last date of contact as reported within the trial (equivalent to Censor date 1 in Table 3). The number of patients for who external data was obtained, by treatment group, will be presented in order to assess the potential for bias in estimates due to more complete and up-to-date survival information being available for a greater proportion of patients in one arm over the other.

Survival data from NHS Digital will not be used to calculate FFS or other progression events.

4.10 POPULATIONS

We define two populations for analysis; the intention-to-treat population and the safety population.

4.10.1.A Intention-to-treat (ITT) population

- Comprised of all randomised patients, whether or not they actually received the allocated trial treatment.
- In ITT analyses by treatment arm, patients will be included in the treatment arm to which they were randomised.

4.10.1.B Safety population

- For the "M1|RT comparison", any patient who was reported as starting radiotherapy within one year after randomisation is included in Arm H-safety in analyses of the safety population.

DocuSign Envelope ID: 67C184DE-98FB-4FA6-BD6F-B91732928013

- Patients for whom radiotherapy treatment was not reported as started within one year of randomisation will be included in Arm A-safety (standard-of-care) in analyses of the safety population.
- Two sensitivity safety analyses will be conducted around this classification, wherein patients with unclear treatment starting status will be included firstly in their allocated treatment arm (making the assumption they start trial treatment) and secondly in the control arm (making the assumption they don't start trial treatment).

The ITT population will be used for all analyses unless specified. The safety population will be included in analyses of adverse events and other safety data (safety analyses).

For visual illustration, a CONSORT flow diagram will clearly identify any patients found to be ineligible post-randomisation or stopping trial follow-up early; these patients will be included in relevant analyses where possible. For reference, a template flow diagram can be found in S:\MRCCTU_Stampede_Stats\SAP.

Prior to the analysis all recorded protocol deviations relating to patients in the "M1|RT comparison" will be reviewed to determine their potential impact on the planned analysis. If substantial issues are found or there are concerns that deviations relating to patient eligibility, treatment, follow-up etc. could affect the validity or interpretation of the results, sensitivity analyses may be performed to evaluate the potential impact. For example, if a substantial proportion of patients are found to be ineligible for the comparison, a sensitivity analysis may estimate the effect of treatment in this subgroup compared to the other patients. The reason for any such sensitivity analyses will be clearly explained when included in the paper and final statistical report.

4.11 SAFETY ANALYSES

Safety analyses will be performed and presented on the safety population; for headline numbers reporting toxicity, these will be repeated on the ITT population.

For the "M1|RT comparison" long-term follow-up analysis, the focus will be on understanding if there are any long-term differences in reporting of urinary, GI and sexual function symptoms.

Safety analyses will focus on adverse events, which sites are expected to report from the time the patient was randomised until disease progression (amended to until 30 days after SOC hormone therapy in the pre-progression setting is discontinued as of Protocol version 19, implemented 26-Nov-2018). Sites are also expected to report serious adverse events (SAEs) that are related or suspected to be related to treatment until 30 days after protocol treatment is stopped. During the period of data collection for the "M1|RT comparison" this has been interpreted differently for patients allocated to arm A and arm H. For control patients information about SAEs experienced until 30 days after the discontinuation of SOC hormone therapy in the pre-progression setting has been recorded, whereas for arm H patients information was only collected about SAEs experienced up to 30 days following the completion of protocol-specified prostate radiotherapy. The time period during which SAEs were recorded for analysis therefore differs between the comparison arms, with this ceasing for

DocuSign Envelope ID: 67C184DE-98FB-4FA6-BD6F-B91732928013

arm H patients undergoing research radiotherapy earlier than for control patients. As events recorded as SAEs are also expected to be reported as adverse events on the standard Follow-Up / Toxicity CRFs and the focus of the analysis will mostly be on the highest grade reported by a patient for a particular event this disparity between the coverage of data collection for SAEs is not expected to result in a biased comparison of adverse events as a whole. Comparative analyses of SAE data alone will acknowledge the potential for bias due to the longer duration of data collection for control patients in the "M1|RT comparison".

Safety will be evaluated by tabulation of adverse events at or up to pre-defined follow-up time points. Adverse events will be classified using the NCI Common Toxicity Criteria for Adverse Events and summarised for each treatment arm. Reported grading is "0 = toxicity not experienced" up to "5 = fatal". CTCAE v4.03 has been used for all assessments dated from Protocol v19.0 (26-Nov-2018) onwards; prior to this, CTCAE v4.0 was used for assessments dating from Protocol v15.0, 05-Sep-2016; assessments made before this used CTCAE v3.0.

In the published results of the primary analysis of the "M1|RT comparison", adverse events were reported using CTCAE v4.0, with assessments made using the earlier CTCAE v3.0 re-coded to fit with CTCAE v4.0. For the long-term follow-up analysis of the comparison, CTCAE v4.0 will be used to facilitate comparison with the earlier analysis. Events assessed using CTCAE v4.03 will be re-coded to fit with CTCAE v4.0.

Adverse events (AEs) may be detected through several sources reported by sites on CRFs:

1. **Follow-up CRF** – routinely reported symptoms and "toxicities" (severity not seriousness reported)
    - AEs reported here up to Sep-2016.
2. **Toxicity CRF** – prompted reporting of symptoms and "toxicities" (severity not seriousness reported)
    - AEs reported here from Sep-2016 onwards.
    - Linked to routine follow-up visits, where sites are asked to report any toxicities experienced in the period covered by the follow-up assessment; and treatment actions and permanent stopping of treatment where toxicity is given as the reason for the action.
3. **SAE CRF** – spontaneously reported serious adverse events (severity <u>and</u> seriousness reported)

Not all serious events are severe nor are all severe events serious.

"Seriousness" is a term specific to the reporting of events to regulatory bodies. We have prioritised the consideration of "severity" for balancing evidence of treatment side-effects against activity data. SAE, Follow-up and Toxicity forms all request the severity of events. Therefore, these sources can be merged to form one dataset for reporting the **severity** of toxicities experienced across different body systems and specific disease categories. The focus of severity-reporting will be on toxicities with grade 3, 4 or 5 (fatal), however all toxicity grades will be reported for completeness.

Reporting windows will be defined around set time points which will be as close to the time of interest while accepting that clinical practice means that most patients will not be reviewed on a specific day. These windows are as follows:

DocuSign Envelope ID: 67C184DE-98FB-4FA6-BD6F-B91732928013

- Toxicity at two years after randomisation: based on information provided for follow-up assessment, Toxicity report or SAE report closest to a patient's 2-year anniversary of randomisation, within 12 weeks of this anniversary. Patients are included in the relevant cross-sectional analysis if they reached 96 weeks since randomisation with reported assessments all without progression. Data on radiotherapy-related toxicities collected at follow-up assessments using RTOG late side-effect gradings around the two year time point will also be presented for these patients.

- Toxicity at four years after randomisation: based on information provided for follow-up assessment, Toxicity report or SAE report closest to a patient's 4-year anniversary of randomisation, within 12 weeks of this anniversary. Patients are included in the relevant cross-sectional analysis if they reached 192 weeks since randomisation with reported assessments without progression. Data on radiotherapy-related toxicities collected at follow-up assessments using RTOG late side-effect gradings around the four year time point will also be presented for these patients.

We will also present toxicity data reported from randomisation **up to** the pre-specified time points above; this will include all patients with follow-up/toxicity/SAE data available within that time frame.

All patients receive ADT as standard-of-care and so interest will be in the additional toxicity reported for patients on research arm relative to control arm, compared informally. Interest will also be in any proportion of known treatment toxicity above that which is expected in this population.

"Relatedness" is only collected for SAEs and cannot be reported for all adverse events.

4.12 MISSING DATA

Missing data on adjustment variables will be imputed using mean imputation in the main intention-to-treat analyses. Information on covariates included as adjustment variables in the main pre-specified analysis models is collected at randomisation so these key variables should be complete in most cases. There is no reason to believe that missingness in these variables is related to any of the outcome measures of interest. Mean imputation is consistent with the assumption that the randomisation process ensures the distribution of adjustment variables is balanced across the trial arms in a comparison.

For subgroup analyses only complete cases will be included. Missing data on the baseline variables used to categorise patients into subgroups will be assumed to be missing completely at random (MCAR) for these analyses.

4.13 PRE-SPECIFIED SUBGROUP EFFECTS

4.13.1 METASTATIC VOLUME

During the trial, interest in metastatic "volume" grew after patients in the CHAARTED trial of docetaxel were divided into "low volume" and "high volume" disease. For the LATITUDE trial of abiraterone metastatic patients

DocuSign Envelope ID: 67C184DE-98FB-4FA6-BD6F-B91732928013

were divided into "low risk" and "high risk" and only the latter were recruited. In the HORRAD trial of radiotherapy for metastatic disease (*European Urology* 75 (2019) 410-418), patients were divided into "oligo-" and "poly-metastatic" subgroups.

In STAMPEDE, volume has been estimated by retrospective collection of bone and CT scans for metastatic patients, and subgroup analysis of treatment effect will be conducted. For the "M1|RT comparison" long-term follow-up analysis, patients will be classified as having "low" or "high" metastatic burden at baseline and analysed accordingly, as per the earlier primary analysis in 2018.

Heterogeneity of treatment effect on survival by baseline metastatic burden will be explored using the metastatic burden x treatment allocation interaction term p-value from an adjusted Cox model or flexible parametric model (see above) with metastatic burden included as a binary explanatory variable, fitted to data from M1 patients for whom volume data is available.

A related analysis will consider the evidence of differing survival in patient groups based on an updated definition of "low" and "high" metastatic burden, as defined in a recent exploratory analysis undertaken by the team at the Christie, published in *JAMA Oncology*[7]. Patients with non-regional lymph node (NRLN) metastases only at baseline or patients with ≤ 3 bone metastases (with or without additional NRLN lesions) will be classified as having "low metastatic burden" disease under this new definition. Patients with > 3 bone metastases (with or without additional NRLN lesions) or with evidence of visceral / other metastases at baseline will be classified as having "high metastatic burden" disease. The same approach will be taken to this analysis as for that based on the earlier definition of low / high burden disease, namely with an interaction test used to assess the evidence in favour of a heterogeneous treatment effect.

4.13.2 Radiotherapy Schedule

For the "M1|RT comparison", a proposed RT schedule was nominated for each patient at randomisation (prior to allocation to Arm H or the control group) with roughly half of patients being nominated to each option. At the time of the primary analysis a time-to-FFS analysis "within schedule" was carried out. A formal test for a difference in treatment effect by planned RT schedule will be performed using p-value for interaction between treatment effect and an indicator for RT schedule, and separate survival analysis models fitted to estimate treatment effect in the two RT schedule groups. At the time of the main analysis in 2018, ~300 control arm FFS events were expected within each RT schedule group, which would give 90% power to detect a proportional treatment difference equivalent to a HR of 0.75 with one-sided alpha = 0.015. If one or both of the RT schedules show evidence of an effect on FFS, a formal comparative "within schedule" analysis will be carried out on overall survival when ~199 control arm deaths are observed in that schedule comparison. This is a closed test, with survival only formally compared within schedule if there is an advantage in FFS observed for that RT schedule at the main analysis. This closed test procedure will be repeated, with limited power, within the volume-defined groups.

[7] Ali A, Hoyle A, Harran H, et al. Association of Bone Metastatic Burden With Survival Benefit From Prostate Radiotherapy in Patients With Newly Diagnosed Metastatic Prostate Cancer: A Secondary Analysis of a Randomized Clinical Trial. *JAMA Oncology* 2021; published online 18-Feb-2021: doi:10.1001/jamaoncol.2020.7857

DocuSign Envelope ID: 67C184DE-98FB-4FA6-BD6F-B91732928013

4.13.3 Stratification Factors

The stratification factors considered at the point of randomisation (except for randomisation centre and method of hormones) will form subgroups in which treatment effect on the primary and secondary outcomes can be assessed, with an interaction p-value of less than 0.05 used to suggest evidence of a difference in treatment effect across the relevant subgroups. As with all subgroups, we accept that there is limited power to detect an interaction and for analyses restricted to patients in a particular subgroup. The raised probability of a type 1 error from multiple testing will be acknowledged. In addition, the time periods the relevant comparison was recruiting across and Gleason score may be considered as subgroups in which to assess treatment effect, along with pre-hormone therapy PSA as a continuous variable.

4.13.4 Other Subgroup Analyses

There are no additional pre-specified comparison-specific subgroup analyses.

4.14 COMPARATIVE ANALYSIS OF QUALITY-OF-LIFE DATA

In addition to assessing adverse event data provided by researchers at sites, we will compare the longitudinal change in patient-reported quality-of-life (QoL) from randomisation over two years and assess if there are significant differences in quality-of-life for patients allocated to SOC treatment alone or SOC + radiotherapy. It was felt that the first two year period after randomisation would provide a comprehensive review of patients' experience of commencing SOC treatment and the period during and following protocol-specified radiotherapy for arm H patients. We will perform separate analyses for patients categorised as having low and high-burden metastatic disease at baseline, according to the CHAARTED definition used earlier.

4.14.1 Data sources

The questionnaires used to collect QoL and related Health Economics (HE) data are:

- EORTC QLQ-30 Quality of life form
- EORTC PR-25 Quality of life form
- EQ-5D Health Economics form

This QoL analysis will only use information collected on the QLQ-30 CRF.

4.14.2 Data completion schedule

The following table gives detail around the expected timing of the scheduled QoL and HE CRFs, as originally planned.

DocuSign Envelope ID: 67C184DE-98FB-4FA6-BD6F-B91732928013

Table 5: Timings for completion of scheduled CRFs

Timing of Assessment		Outcomes	
		QoL, HE CRFs	Freq
Yr 0	Wk 0	all	
	Wk 6	all	6-weekly
	Wk 12	all	
	Wk 18	all	
	Wk 24	all	
	Wk 36	all	12-weekly
	Wk 48	all	
	Wk 60	all	
	Wk 72	all	
	Wk 84	all	
	Wk 96	all	
Yr 2	Month 24	all	6-monthly
	Month 30	all	
Yr 3	Month 36	all	
	Month 42	all	
Yr 4	Month 48	all	
Yr 5	Month 60	all	
Yr 6+		all	Annual

Since the implementation of Protocol version 15 from 05-Sep-2016, the planned collection of QoL and EQ-5D data was modified so that patients were expected to provide this information at each scheduled assessment until the earliest of disease progression or 5 years of follow-up.

From the implementation of Protocol version 19 on 26-Nov-2018, further collection of QoL and EQ-5D data was ceased in all patients randomised to the "M1|RT comparison" prior to 01-Apr-2016, regardless of progression status or time on trial, and only EQ-5D data was collected for patients randomised to the comparison from 01-Apr-2016 to 02-Sep-2016. From the implementation of Protocol version 21 on 30-Nov-2020, collection of EQ-5D data in the remaining patients randomised to the comparison between 01-Apr-2016 and 02-Sep-2016 ceased.

4.14.3 DATA VERIFICATION

Problems with QoL and HE data have not been routinely queried with sites. Handling of missing data is described below.

4.14.4 COVERAGE OF ANALYSIS

Analyses will be based on all QLQ-30 CRF data that was received by the CTU for patients in the "M1|RT comparison" up to the data freeze taken for the primary analysis on 01-Jun-2018. Patient responses will be censored at the point of progression (with no further observations after this point included in statistical models). This may limit the data included in the analysis for a proportion of patients randomised at a relatively early point to the comparison, who may have continued to provide QoL data following progression. For many patients randomised close to the end of recruitment to the comparison, the updated guidance in Protocol version 15 (05-Sep-2016) that QoL data should be provided up to progression is expected to have come into effect prior to the

DocuSign Envelope ID: 67C184DE-98FB-4FA6-BD6F-B91732928013

patient experiencing disease progression; consequently, no post-progression data is expected from these patients.

4.14.5 POPULATIONS

We define the population for the QoL analysis as the intention-to-treat population who have consented to QoL data collection. This comprises all applicable randomised patients, whether or not they actually received their allocated trial treatment. All patients will be included in the treatment arm to which they were randomised.

For visual illustration, a CONSORT flow diagram will clearly identify any patients found to be ineligible post randomisation or stopping trial follow up early; these patients will be included in relevant analyses where possible.

4.14.6 MISSING DATA

Missing data are a common problem in QoL analyses. The assumption of missing at random or missing completely at random for expected but unobserved observations is unlikely to hold since patients may not be asked to complete CRFs if they are feeling unwell, or if they have recently been informed that their disease has progressed. Observations are also truncated due to death.

The amount of expected but missing data will be reported for each arm. If required, sensitivity analyses will be conducted to evaluate the effect of missing CRFs. For this, missing data for patients who are still alive will be imputed and a variety of scenarios will be tested, providing an indication of the robustness of results.

4.14.7 MAIN OUTCOME MEASURE FOR QUALITY-OF-LIFE ANALYSIS

Global Quality-of-Life ("Global QoL") as a percentage will be derived from answers to questions 29 and 30 on the QLQ-30 CRF ('how would you rate your overall health during the past week?' and 'how would you rate your overall quality of life during the past week?'). Lower patient-reported grading for these questions will correspond to a lower Global QoL %, with a patient reporting both their overall health and quality of life as 1 = "very poor" equivalent to 0% and a patient reporting both as 7 = "excellent" equivalent to a Global QoL of 100% for that observation. Patients with only one of questions 29 and 30 answered will have a Global QoL value calculated based on the assumption that the other question would have been answered with the same score.

Difference in average Global QoL between patients allocated to arm A and arm H will be assessed over the course of the first two years following randomisation. This will be evaluated using longitudinal analysis of Global QoL self-assessment scores using a "partly-conditional" or "while-alive" approach.

We will use multivariate imputation using chained equations with predictive mean matching to impute missing observations on Global QoL at each scheduled time point. Each chained equation will include as predictors observed values of the outcome at all other time points, the baseline stratification factors used in the main survival analysis models for this comparison (nodal stage, regular aspirin/NSAID use, WHO performance status [0 vs 1+], age at randomisation [<70 vs 70+]), and other baseline observations that are potentially predictive of subsequent quality of life: metastatic burden and pain from prostate cancer. We will impute missing values separately for the two treatment groups. We will modify the specification of the chained imputation process if

DocuSign Envelope ID: 67C184DE-98FB-4FA6-BD6F-B91732928013

problems with model convergence arise. Using this process we will create several imputation datasets, with the number at least equal to the percentage of missing observations at the least well-reported time point, in line with previously published guidance (*Statist. Med.* 2011, 30 377–399). The number of iterations to be performed will be guided by checks to ensure convergence has been reached, using trace plots for example. Imputed observations on global QoL that were originally missing due to the patient having died prior to the scheduled assessment will be set to missing again.

Using the imputation datasets we will model expected global QoL in each treatment group at each scheduled time point using generalised estimating equations with an independence correlation structure. An interaction term for treatment x time will be specified for each time point. This will estimate the mean global QoL at a given time for each treatment group separately in patients who were alive at that point (a "survivors analysis"). The pooled model-fitted expected Global QoL % for all time points will be used to calculate a weighted average for Global QoL over the first two years on trial for each treatment group (with weighting corresponding to the length of follow-up time covered by each follow-up assessment, e.g. 6 weeks for each observation up to and including week 24). A Wald test will be used to assess whether there is evidence of a difference in this weighted average between the two groups, using the `-lincom-` postestimation command in Stata. Any differences seen between the two treatment groups will need to be assessed with reference to the results from survival modelling, as this analysis will only reflect patient-reported quality-of-life in patients who are still alive at each scheduled time point.

An alternative longitudinal analysis of Global QoL will use a "composite" outcome that combines patient-reported information and survival status. Observations that are missing due to a patient having died prior to the scheduled time point will be defined as being equivalent to a Global QoL score of 0%, the lowest possible score. Other observations, including missing assessments from when a patient was alive, will be left unchanged. A linear regression model will be fitted to this modified data with patient-level random intercept and a separate covariate at each scheduled follow-up assessment time point, each with an interaction term allowing for a difference in average Global QoL according to trial arm at that time point. The model will also include a patient-level random slope effect that allows the change in Global QoL over time to vary between patients. Estimation of the fixed effects and an unstructured 2x2 variance-covariance matrix for the random intercept and slope will use restricted maximum likelihood (REML) techniques.

As before, the expected Global QoL % for all time points as estimated by the mixed model will be used to calculate the difference in (weighted) average between the treatment groups. The estimand for this analysis is the difference in expected Global QoL score over time between treatment policy groups in all patients, with Global QoL equivalent to 0% at all assessments after a patient has died.

Details of these two analytical approaches and the estimands they are targeting are included in Table 6.

4.14.8 SECONDARY OUTCOME MEASURES FOR QUALITY-OF-LIFE ANALYSIS

4.14.8.A Cross-sectional comparison of Global QoL

We will assess the difference in expected Global QoL at 12 weeks, 24 weeks, 60 weeks and 2 years between the treatment groups. These will be cross-sectional analyses, adjusted for baseline Global QoL score, using only data

from patients who survived beyond the time point of interest and who provided sufficient data for a Global QoL score to be determined for that time. A linear regression model will be fitted with Global QoL at the time point of interest specified as the outcome, a binary predictor corresponding to treatment allocation and an additional covariate for baseline Global QoL %. The results will be used to assess whether there is evidence of a difference between the two patient groups.

Details of this cross-sectional analytical approach and the estimand it is targeting are included in Table 6.

4.14.8.B Cross-sectional and longitudinal comparison of QoL Summary Score

QLQ-30 Summary Score as a percentage will be derived from answers to questions 1-27 in the QLQ-C30 (comprising 13 scales, but excluding questions 28-30 relating to the financial scale and global quality-of-life). This is a validated summary score supported by EORTC. It will be calculated for a given assessment time point for a patient only if more than half of the questions corresponding to each scale have been answered. The relevant functional and symptom scales, along with the minimum number of questions that require to be answered, are as follows:

- Physical functioning (questions 1-5): ≥ 3 questions answered
- Role functioning (6, 7): both questions answered
- Emotional functioning (21-24): ≥3 questions answered
- Cognitive functioning (20, 25): both questions answered
- Social functioning (26, 27): both questions answered
- Fatigue (10, 12, 18): ≥ 2 questions answered
- Pain (9, 19): both questions answered
- Nausea & vomiting (14, 15): both questions answered
- Dyspnoea (8): question must be answered
- Sleeping disturbances (11): question must be answered
- Appetite loss (13): question must be answered
- Constipation (16): question must be answered
- Diarrhoea (17): question must be answered

For each of the functional / symptom scales above, a percentage score is calculated based on the answers to the component questions. For both functional scales, a lower numerical response represents the best possible state for a patient (e.g. '1' for each of question 1 – 5 in the 'physical functioning' scale) and a higher numerical

response represents the poorest possible state (e.g. '4' for each question in the same 'physical functioning' scale). These numerical responses are scaled to a percentage, with 0% representing the best possible state (the lowest numerical response) and 100% representing the poorest possible state (the highest numerical response). Summary Score will be calculated as the equal-weighted mean of the percentage scores associated with each individual scale, with a higher Summary Score percentage corresponding to poorer function and worse symptoms.

We will perform longitudinal analyses of Summary Score over the first two years on trial using the partly-conditional approach outlined above, and a composite outcome where Summary Score is assumed to be 100% (equivalent to the poorest function and worst symptoms) at any assessment after a patient has died. We will also assess the difference in expected Summary Score at 12 weeks, 24 weeks, 60 weeks and 2 years between the treatment groups using data from complete cases (i.e. those who were alive and provided sufficient information for Summary Score to be determined at the relevant time point).

Table 6: Definition of estimands in quality-of-life analyses

Outcome	Analysis	Estimator	Analysis assumptions	Relevant inter-current events	Data source	Estimand
Patient-reported Global QoL %	Partially-conditional, GEEs with independence working matrix	Difference in weighted average of outcome in first two years of trial between treatment groups	Missing observations in patients who are alive are missing at random, conditional on observed data	Death (truncated observations)	Observed and multiply-imputed outcome data for survivors; no observations after a patient has died	Difference in average Global QoL in first two years following diagnosis of high-risk prostate cancer due to treatment policy of upfront radiotherapy, in patients who are alive
Patient-reported Global QoL %	Composite outcome, mixed effects linear regression with potentially-correlated random intercept and slope	Difference in weighted average of outcome in first two years of trial between treatment groups	Patient-level Global QoL % at different time points is correlated according to random effects specification; missing observations in patients are missing at random,	None	Observed and implicitly imputed outcome data for survivors; post-death observations defined as 0% Global QoL	Difference in average Global QoL in first two years following diagnosis of high-risk prostate cancer due to treatment policy of upfront radiotherapy,

			conditional on observed data			in patients who are alive or dead, with Global QoL 0% after death
Patient-reported Global QoL %	Cross-sectional, baseline-adjusted linear regression	Difference in change in expected outcome from baseline between treatment groups	Missing observations in surviving patients are missing completely at random	Death (truncated observations)	Complete cases alive at associated time point	Difference in average Global QoL in patients who are alive at a specific time following diagnosis of high-risk prostate cancer
Patient-reported QLQ-30 Summary Score (%)	Partially-conditional, GEEs with independence working matrix	Difference in weighted average of outcome in first two years of trial between treatment groups	Missing observations in patients who are alive are missing at random, conditional on observed data	Death (truncated observations)	Observed and multiply-imputed outcome data for survivors; no observations after a patient has died	Difference in average Summary Score in first two years following diagnosis of high-risk prostate cancer due to treatment policy of upfront radiotherapy, in patients who are alive
Patient-reported QLQ-30 Summary Score (%)	Composite outcome, mixed effects linear regression with potentially-correlated random intercept and slope	Difference in weighted average of outcome in first two years of trial between treatment groups	Patient-level Summary Score % at different time points is correlated according to random effects specification; missing observations in patients are missing at random, conditional on observed data	None	Observed and implicitly imputed outcome data for survivors; post-death observations defined as 0% Global QoL	Difference in average Summary Score in first two years following diagnosis of high-risk prostate cancer due to treatment policy of upfront radiotherapy, in patients who are alive or dead, with Summary Score 100% after death
Patient-reported QLQ-30	Cross-sectional, baseline-adjusted	Difference in change in expected	Missing observations in surviving patients are	Death (truncated observations)	Complete cases alive at	Difference in average Summary Score in

DocuSign Envelope ID: 67C184DE-98FB-4FA6-BD6F-B91732928013

Summary Score (%)	linear regression	outcome from baseline between treatment groups	missing completely at random		associated time point	patients who are alive at a specific time following diagnosis of high-risk prostate cancer

4.14.9 MINIMALLY IMPORTANT DIFFERENCE

To determine if any difference in average Global QoL observed between the treatment groups in the first two years on trial (using data from survivors at each time point) is clinically significant we will use the following categorisations, primarily informed by a 2011 paper by Cocks et al (*J Clin Oncol* 29:89-96) and a 1998 paper by Osabo et al (*J Clin Oncol* 16:139-144). If the 95% confidence interval around the point estimate for the difference in weighted averages does not include values in the range 0-4 we will conclude that there is evidence of a clinically significant difference.

Table 7: Classification of clinical relevance of observed difference in Global QoL

**Effect size**	**difference in % score**
Trivial (unlikely to have clinical relevance or no difference)	0-4
Small (subtle but none-the-less clinically relevant difference)	>4-10
Medium (likely to be clinically relevant but to a lesser extent than a large effect)	>10-15
Large (obvious and unequivocally clinically relevant)	>15

DocuSign Envelope ID: 67C184DE-98FB-4FA6-BD6F-B91732928013

5 ANALYSIS DETAILS

The results of the analyses will be reported following the principle of the ICH E3 guidelines on the Structure and Content of Clinical Study Reports.[8]

For the final efficacy analyses, the flow of patients through the trial during the time the relevant comparison was recruited will be presented in a CONSORT diagram.

5.1 PRE-PLANNED DATA CHECKS

- Disease progression reported on FU form *vs* progression form; check for concordance
- Death reported on progression form *vs* death form [deaths reported before 05-Sep-2016]; check for concordance
- Death reported as an SAE on Death CRF *vs* SAE form with death reported
- Observed PSA at failure as reported on progression form *vs* expected minimum PSA value at failure and sequential PSA values reported at follow-up assessments

5.2 PLANNED ANALYSES

5.2.1 BASELINE CHARACTERISTICS

Although previously reported, the following baseline characteristics will be presented, broken down by treatment arm unless otherwise stated, either as n (%) or median (IQR; min-max):

- All stratification factors
- Randomisation CRF data:
    - Age at randomisation (years)
    - PSA at randomisation (ng/ml; defined as PSA pre-HT) – log transformed
    - Time from diagnosis to randomisation (days)
    - Pain from prostate cancer at randomisation: Absent; Present
    - Broad disease category: N0M0 new; N+M0 new; M1 new; Local treatment now relapsing (all patients in the "M1|RT comparison were randomised on the basis of having newly-diagnosed metastatic disease at the time of randomisation")
    - T-stage at randomisation
    - N-stage at randomisation
    - Any metastases at randomisation (all patients in the "M1|RT comparison were randomised on the basis of having metastatic disease at the time of randomisation")
    - Bone metastases at randomisation
    - Liver metastases at randomisation
    - Lung metastases at randomisation
    - Nodal metastases at randomisation
    - Other metastases at randomisation
    - Metastatic volume (when available)

[8] http://www.ich.org/fileadmin/Public_Web_Site/ICH_Products/Guidelines/Efficacy/E3/E3_Guideline.pdf

DocuSign Envelope ID: 67C184DE-98FB-4FA6-BD6F-B91732928013

  - Use of aspirin
  - Use of NSAIDs
  - Use of short-term bisphosphonates
  - Planned type of HT (as reported on Randomisation CRF)
  - Planned use of long-term anti-androgens
  - Participation in QL study
  - Time from randomisation to starting current HT (times negative if pt starts HT pre-randomisation)
  - Previous HT type: None; LHRH (agonist or antagonist if known); AAs alone; DAB [patients should all be newly-diagnosed]
  - Previous local therapy type (if known): None; radical prostatectomy; radical radiotherapy; radical prostatectomy with post-operative radical radiotherapy; other [pts should all be newly-diagnosed]
  - Duration of previous HT (days) [pts should all be newly-diagnosed]
  - Months between end of previous HT and randomisation (subgroup: broad disease category) [pts should all be newly-diagnosed]
- Baseline CRF data:
  - Gleason sum score at presentation
  - T-stage at presentation
  - N-stage at presentation
  - PSA at first presentation (ng/ml) – log transformed
  - Concomitant medications (to be clinically recoded)
- Cardiovascular assessment data
  - Smoking status
  - Diabetes and type
  - History of MI, CV disease, CHF, angina or hypertension

5.2.2 STANDARD-OF-CARE TREATMENT

Only if additional relevant data have been received since the main analysis, for all standard-of-care treatments the following data will be presented, broken down by treatment arm:

- Hormone therapy details (from the FU CRF, HT CRF or the SOC HT Log)
  - Numbers reporting changing or stopping treatment (N, %)
  - Time to treatment action
  - Reason for treatment action
- Docetaxel treatment details (from the SOC Docetaxel Treatment CRF; planned details from Randomisation CRF)
  - Reported vs planned docetaxel
  - Time from randomisation to first cycle of docetaxel (days)
  - Time from starting ADT to first cycle of docetaxel (days)
  - Number of cycles administered
  - Reason for less than 6 cycles

DocuSign Envelope ID: 67C184DE-98FB-4FA6-BD6F-B91732928013

  - Daily steroid formulation
  - Daily steroid dose

5.2.3 TRIAL TREATMENT

Only if additional relevant data have been received since the main analysis, for **Arm H radiotherapy** treatment data the following analyses are planned:

- Number of patients starting RT
- Details of patients confirmed as not receiving RT
- Time from randomisation to first fraction of RT (in all pts; censor those who don't report starting)
- Planned vs reported RT schedule (planned details on the Randomisation CRF)
- Details of patients with different RT dose reported to that planned
- Graph of planned vs reported RT dose
- Details of patients with RT reported as being delayed
- RT treatment details; time to stopping (days from randomisation) and reason for stopping
- Graph of time from randomisation to last RT fraction
  - Censor at randomisation if explicitly reported not having RT as allocated
  - Censor at last contact if RT not yet reported
- Graph of duration of RT (first to last RT fraction; in pts who started)
- Details of patients who report RT to the pelvic nodes

For both treatment arms:

- Additional treatments given (as reported on the Additional Treatment CRF)
  - Include detail of any patients receiving non-protocol disease-directed interventions prior to study outcomes

5.2.4 TRIAL EVENTS

All trial outcomes will be analysed on an ITT basis, as per randomised allocation regardless of treatment received.

5.2.4.A Primary Outcome Measure

For the primary outcome of overall survival, the following data will be presented:

- Incidence of death, by treatment arm
- Estimates of survival over time from randomisation, focusing on 1, 3 and 5 years, by treatment arm (%, 95% CI)
- Cause of death, by treatment arm (as determined using "M1|RT comparison"-specific process)
- Death within 4 weeks of administration of trial treatment, by treatment arm
- Death related to trial treatment, by treatment arm
- Time from randomisation to death from any cause, by treatment arm
  - Test for difference in survival using log-rank test stratified across baseline stratification factors (excluding centre and method of hormones) plus the time periods covered by recruitment to the "M1|RT comparison"
  - KM survival plot (in KMunicate format)

DocuSign Envelope ID: 67C184DE-98FB-4FA6-BD6F-B91732928013

  - Censor individuals at last contact if not died
- Grambsch-Therneau test for proportional hazards assumption using log-transformed time, based on Schoenfeld residuals from fitting a Cox model adjusted for applicable stratification factors and stratified by relevant time periods
- Estimation of hazard ratio for treatment effect over time using FPM with time-dependent treatment effect, adjusted for applicable stratification factors and time periods
  - Suitable t-star to be calculated at time of analysis
- Explicit statement of whether adjusted Cox model or RMST takes primacy
- Calculation of restricted mean survival time using FPM with time-dependent treatment effect
- Test for heterogeneity of treatment effect across stratification factors, Gleason score and pre-HT PSA as continuous variable
- The following sensitivity analyses will be undertaken for comparisons of research vs control, with model-estimated hazard ratio for treatment effect and 95% CI reported:
  - Log-rank HR (stratified)
  - Unadjusted Cox model
  - Flexible-parametric model with time-fixed covariates
  - Adjusted Cox with time-dependent WHO performance score

5.2.4.B Secondary Outcome Measures

For all secondary outcomes the following analyses will be performed:

- Incidence of the outcome, by treatment arm
- Estimate of (*freedom from*) outcome over time from randomisation, focusing on 1, 3 and 5 years, by treatment arm (%, 95% CI)
- First reported event, by treatment arm [FFS only]
- Time from randomisation to outcome, by treatment arm
  - Test for difference in survival using log-rank test stratified across baseline stratification factors (excluding centre and method of hormones) plus the time periods covered by recruitment to the "M1|RT comparison"
  - KM survival plot
  - Censor individuals at last contact if outcome not reported
- Estimation of hazard ratio for treatment effect over time using FPM with time-dependent treatment effect, adjusted for applicable stratification factors and time periods
  - Using t-star determined for primary outcome analysis
- Calculation of restricted mean survival time using FPM with time-dependent treatment effect

5.2.4.C Additional analyses by baseline metastatic burden subgroup

For a subset of patients with information on metastatic disease burden at baseline (high/low burden, following the CHAARTED definition), the following analyses will be performed for the primary outcome measure of OS:

- Incidence of death, by treatment arm, within high/low burden subgroup
- Time from randomisation to death from any cause, by treatment arm, within high/low burden subgroup:
  - KM survival plot

- Median survival time estimated from FPM
- Censor individuals at last contact if outcome event not reported

- Test for differential treatment effect by burden of metastatic disease, using interaction term p-value from adjusted Cox model with metastatic burden included as an additional binary explanatory variable
- Comparison of research vs control from adjusted Cox model, within high/low burden subgroup
- These analyses will also be performed for high/low metastatic burden subgroups, as per the updated definition proposed by researchers at the Christie

5.2.4.D Additional analyses by intended RT schedule subgroup

We will perform the following analyses of the primary outcome of OS within subgroups defined by which RT schedule a patient was planned for prior to randomisation:

- Incidence of death, by treatment arm, within intended RT schedule subgroup
- Time from randomisation to death from any cause, by treatment arm, within intended RT schedule subgroup:
    - KM survival plot
    - Median survival time estimated from FPM
    - Censor individuals at last contact if outcome event not reported
- Test for differential treatment effect by intended RT schedule, using interaction term p-value from adjusted Cox model with intended RT schedule included as an additional binary explanatory variable
- Comparison of research vs control from adjusted Cox model, within intended RT schedule subgroup

5.2.5 Toxicity/Safety

Toxicity data will be reported using the NCI Common Toxicity Criteria for Adverse Events and are to be presented in the **safety population for final analysis publication and reports**, with only headline figures shown for the ITT population to demonstrate comparability of the populations.

Data presented by treatment arm should be:

- KM plot of time to first G3-5 toxicity reported on the FU, Toxicity or SAE CRF; include maximum SAE grade
- Worst toxicity grade in any category (overall & subgroup: metastatic status); in categories relating to urinary, bowel and sexual function; proportion with grade 3-5, for the following time points/periods:
    - At 2 years (+/- 12 weeks)
    - From randomisation up to 2 years
    - At 4 years (+/- 12 weeks)
    - From randomisation up to 4 years
    - Ever on trial
- Time to first grade 3-5 SAE
- Time to any grade SAE
- Time to first grade 3-5 SAR
- Time to any grade SAR

- Time to first grade 3-5 SUSAR
- Time to any grade SUSAR

[Given that the SAE collection period can vary by arm, sensitivity analyses will cap SAE-based analyses at 2 years after randomisation.]

For Arm H patients the following additional RT late toxicity data will be presented (data collected on Follow-Up CRF):

- Table of worst grade ever reported for each side effect, split by intended dose
- Worst grade reported for each side effect at 2 and 4 years (cross-sectional analysis, with comparative data from arm A patients provided for context)

5.2.6 Data Returns

- Death, and end of trial participation forms received for the control arm
- FU forms expected vs number of FU forms received, by treatment arm
- Scatter ("balloon") plot of timing of most recent FU assessment received for each patient still being followed up at time of comparison closure on 30-Nov-2020 *vs* time from randomisation, by treatment arm (including detail on number of patients randomised, number of deaths, number of withdrawals, number okay and number late for follow-up)
- Duration of follow-up after randomisation (time from randomisation to last contact), by arm, with median estimated using reverse KM plot
- Details of patients recorded as being lost-to-follow-up or for whom early stopping of follow-up and data collection has been reported

5.2.7 Quality-of-life analyses

All QoL analyses will be performed for the full comparison sample and within high/low metastatic burden subgroups, following the CHAARTED definition.

Results of main comparative longitudinal analysis of Global QoL and QLQ-30 summary score across 2 years:

- Number of QLQ-30 CRFs expected and received at each scheduled follow-up assessment time point
- Estimated difference in weighted average Global QoL score and Summary Score within 2 years from baseline between arm H patients and control patients, from "partially conditional" and "composite outcome" analyses.
- Graphs of model-estimated expected global score and summary score over time for both treatment groups, from each analysis
- For interpretation, a difference of 0-4 points will be regarded as trivial, >4-10 points as small, >10-15 points as medium, and >15 points as large.

Results from comparative cross-sectional analyses of Global QoL and QLQ-30 Summary Score using data from complete cases only:

- Estimated difference in change in expected Global QoL score and Summary Score associated with allocation to research treatment from cross-sectional analyses at 12 weeks, 24 weeks, 60 weeks and 2 years.

DocuSign Envelope ID: 67C184DE-98FB-4FA6-BD6F-B91732928013

5.3 EXPLORATORY ANALYSES

Exploratory analyses will be clearly noted as such in all reports and presentations.

These will not be predetermined but will be driven by data emerging from within the trial or from external sources. They may include a look at consistency of treatment effect by centre or centre size; the relationship of baseline characteristics to toxicity and toxicity to outcomes; or the relationship of baseline characteristics to the "missingness" of later data points.

DocuSign Envelope ID: 67C184DE-98FB-4FA6-BD6F-B91732928013

6 SIGNATURES OF APPROVAL

Date: 01-Mar-2021

Version: 1.0

Signatures

Name	Trial Role	Signature	Date
Nick James	Chief Investigator*<br>CCI: "Abiraterone comparison"	DocuSigned by: Nicholas James C376081600384F4...	11-Mar-2021
Chris Parker	CCI: "M1|RT comparison"	DocuSigned by: Chris Parker F69CDC77FC4A4FD...	02-Mar-2021
Matt Sydes	Senior Statistician (unblinded)	DocuSigned by: 6E223845835C4EC...	01-Mar-2021
Adrian Cook	Senior Statistician (unblinded)	DocuSigned by: Adrian Cook 81F44B750145464...	05-Mar-2021
Chris Brawley	Statistician	DocuSigned by: Chris Brawley ECD39730E3DC4D3...	01-Mar-2021
Laura Murphy	Statistician	DocuSigned by: Laura Murphy 1F26FDC94B1A4A1...	01-Mar-2021
Max Parmar	Statistician, CTU Director	DocuSigned by: Max Parmar 5C6D329801D0437...	11-Mar-2021

*On behalf of the STAMPEDE Trial Management Group

Credits: Other statisticians involved in the development of this SAP have been Rachael Jinks, Gordana Jovic, Melissa Spears, Fiona Ingleby and Tra My Pham

Reviewers: Ian White (Feb-2021), Adrian Cook (Feb-2021), Matt Sydes (Feb-2021), Babak Oskooei-Choodari (Apr-2018)
