# Supplementary material for: Radiotherapy to the prostate for men with metastatic prostate cancer in the UK and Switzerland: Long-term results from the STAMPEDE randomised controlled trial
Source: PLoS Med. 2022 Jun 7;19(6):e1003998. doi: 10.1371/journal.pmed.1003998 (PMC9173627; doi:10.1371/journal.pmed.1003998)
Supplement: Attachment — Submitted filename: Response-to-reviewers_2022-02-28_v1.pdf [file pmed.1003998.s016.pdf]

STAMPEDE "M1|RT COMPARISON" – LONG-TERM FOLLOW-UP

Response to reviewers

(Received 07-Feb-2022; Due back 28-Feb-2022)
Version 1.00, 28-Feb-2022

COMMENTS	RESPONSE
**Reviewer #1**	
Parker et al present the updated results of the STAMPEDE trial, arm H. This trial randomized >2000 men with newly diagnosed metastatic prostate cancer in the UK and Switzerland to standard of care (lifelong androgen deprivation therapy) +/- radiation therapy (RT) to the prostate. After the start of enrollment, it was determined that results would be analyzed by the volume of metastatic disease. The authors had previously reported in 2018 that prostate RT improves survival in men with low-volume disease but not high-volume disease, with a significant interaction (Parker et al, Lancet 2018). In this updated analysis, they demonstrate that the survival benefit differences found previously persist with additional follow-up. Furthermore, they present data on local events in the forms of Local Intervention Free Survival and Symptomatic Local Event-Free Survival.	(Not comment required)
This is an important update of the STAMPEDE trial, arm H, which helped change the treatment paradigm for men with low-volume de novo metastatic prostate cancer. While the updated survival findings, which demonstrated the continued benefit of prostate RT in low-volume disease, are not surprising, the authors do provide additional results in terms of local event analyses, toxicity including from RT, and quality of life (QOL) metrics. The findings of this study are important for the general medical public given the prevalence of prostate cancer. The manuscript is generally well-written and presents appropriate analyses.	(Appreciated)

COMMENTS	RESPONSE
**Major comments**	
Local Intervention Free Survival (LIFS) was defined as "consisting of time from randomisation to the first report on case report forms of TURP, ureteric stent, surgery for bowel obstruction, urinary catheter, nephrostomy, colostomy, death from prostate cancer" (lines 120-122). I am not confident that death from prostate cancer should be considered in the definition of LIFS, since death from prostate cancer is uncommonly a consequence of local progression but rather typically due to an increase in metastatic disease burden. Did the authors consider an analysis of LIFS using a definition which excludes death from prostate cancer, using death from any cause as a competing risk event? Similarly, did the authors consider an analysis of Symptomatic Local Event-Free Survival (SLEFS) using a definition that excludes death from prostate cancer, again using competing risks survival analysis? Understandably, the sample sizes for these analyses are likely to be small since it appears that the majority of patients did not have events besides death from prostate cancer be reported.	We are grateful to the reviewer for raising this this. We have taken the opportunity to include these sensitivity analyses, and have referred to these from the Methods and Results (Supplementary Table S5, renumbering others); for space, we haven't quoted this text here. These do not impact the interpretation.
The authors found that when stratified by RT schedule, the hazard ratio (HR) for weekly RT was 1.00 and for daily RT was 0.83 (interaction p=0.088). They then looked at specifically at patients with low-volume status and found a HR of 0.67 for weekly RT and 0.62 for daily RT (interaction p=0.732), demonstrating that RT schedule does not appear to influence outcomes for low-volume patients. As additional exploratory analysis, it may be informative to see a similar analysis of HRs by fractionation schedule for high-volume patients.	For the reviewer, the results within high volume patients are:<br>Weekly subgroup – Treatment HR = 1.216<br>Daily subgroup – Treatment HR = 1.019. Interaction p = 0.232.<br>We do not think this should be included in the manuscript.
One possible confounder in this study is the receipt of additional therapies not reported here, namely novel androgen receptor signaling inhibitors (ARSI). It is possible that patients in one group were more likely to have received ARSIs which can influence multiple outcomes, including survival, local events, toxicities, and quality of life metrics. Are the authors able to provide data on use of these novel therapies and if not, they should discuss this as a limitation of the study.	We have included as Supplementary Figures S5A and S5B time to novel androgen receptor signalling inhibitors (ARSI) by allocated treatment. Figure S5A is time from randomisation in STAMPEDE in all patients; Figure S5B is from a failure-free survival events in only those patients with a recorded FFS event. We find it helpful to present this in both ways and have used this approach elsewhere in the trial.
**Minor comments**	
Line 155: There is an extra period after "criteria."	Corrected

COMMENTS	RESPONSE
In Figure 1, it is unclear to me how the n's in the Long-term analysis row were derived from the Follow-up row, e.g. for the first column, 333+74+609=1016, yet the diagram states that n=1029 were analyzed for efficacy. Can the authors clarify this?	The number of patients with current follow-up is standardly calculated only in patients who were last known to be alive and who have not stopped follow-up early i.e. those patients for whom we still expect follow-up. The number of patients who stopped follow-up early was highlighted in Figure 1, but we have made this clearer.
It might be helpful to relabel metastatic disease burden as "low" and "high" in Table 1 to be uniform in the terminology.	We have made this change, although we had chosen "lower" and "higher" because there are alternative definitions.
The legend descriptions for Figures 2A and 2B appear flipped (lines 210-218). There is no legend for Figure 2C.	We are not seeing the error the reviewer highlights. Perhaps the editor could advise?
The authors state that they performed "cause-specific and competing-risk analyses." They should clarify whether this was done using Cox proportional hazard regression vs Fine-Gray regression and define what the competing risks were. Additionally, for prostate cancer-specific mortality, SLEFS, and LIFS, it would be helpful to see interaction p-values as well.	We have updated the Methods section to clarify that Cox models used for cause-specific analyses and Fine and Gray for competing-risks.<br>We have reported interaction p-values for SLEFS & LIFS as a footnote to Table 2 but we would be very happy not to present them – we are not sure they are helpful.
The authors should clarify the percentage of patients who received 3D conformal RT (3D RT) vs intensity-modulated RT (IMRT). My sense is that the majority of patients likely received 3D RT which, in combination with the generous margins used (8mm posterior and 10mm elsewhere), is expected to increase the level of toxicity from RT compared to more modern, albeit expensive, techniques (i.e. image-guided IMRT with tighter margins, usually around 5mm if not less) used in many other parts of the world. This complicates the interpretation of toxicity and quality of life data presented here and hence should be discussed as a limitation.	For the reviewer, the RT technique was reported by sites as:<br>- Conformal: 276 (36%)<br>- IMRT: 493 (64%)<br>- Missing: 203<br><br>We see this as indicative rather than definitive reporting and do not think this should be included in the manuscript.
**Reviewer #2**	
This paper reports on the long term overall survival, quality of life and local events in patients with primary metastatic prostate cancer, randomised between with radiotherapy to the prostate or standard of care in the SRAMPEDE trial. This is an essential trial since it altered the guidelines into advising radiotherapy to the prostate in patients with low volume metastatic disease. The presented update on the survival data that confirms previous findings is very important. Also reporting on local problems and quality of life is useful, as local radiotherapy of the prostate is known to have a substantial impact on patients' health-related-quality-of-life potentially. The manuscript is well written and has a clear message.<br>There are a few issues in this report that should be addressed.	(No response required)

COMMENTS	RESPONSE
1) In the introduction, the authors write that they report on data on freedom from local interventions and mention urinary catheter, ureteric stents, nephrostomies, colostomy. Why did the authors eventually include death in their analysis on symptomatic local event? This is already addressed in their survival analysis. It there a difference in time to local event or proportion of local events when death is not included in this analysis?	We are grateful to the reviewer for raising this issue, which we have addressed in the response to Reviewer 1 (see above). The message remains unchanged.
2) Table 3 misses in the manuscript.	This was accidentally moved to Table S4. We have reinstated it, but will take editorial advice on whether it would be better in the Supplement.
3) In the introduction, the authors write that the first report on quality of life of this trial. The HORRAD group also published results on the effect on HrQoL of radiotherapy in patients with metastastic prostate cancer. It would be helpful to compare the results of both similar trials in the discussion.	We appreciate this suggestion. We have added to the Discussion:<br>*"Prostate radiotherapy did not have any long-term impact on quality of life either in this trial, or in the HORRAD trial.[11] The risk of toxicity from prostate radiotherapy, although low, could be further reduced by the use of more contemporary intensity modulated techniques.[12]"*<br>This includes a new reference.
4) In analysing QoL questionnaires, only the global QoL scores were presented. Were there any differences in the different domains of the questionnaires between the two groups?	We include QLQ-30 summary score, which is derived from 13 of the functional / symptom domains. We did not plan to analyse individual domains, but someone may wish to propose this as a collaborative data re-use project after publication.
5) For time-to-event variables, report the number of events but not the proportion. Avoid reporting the percentage of deaths in reporting on survival. Although it is important to report the number of events, patients entered the study at different times and were followed for different periods; hence, the reported proportion is meaningless.	We agree this is confusing and have removed mention on % with the numbers of events for time-to-event outcome measures.
**Reviewer #3**	
This is a useful and well-conducted RCT on the long-term results of radiotherapy on the prostate for men with metastatic prostate cancer. The study design, statistical methods and analyses, and presentation (tables and figures) and interpretation of the results are mostly adequate and of a good standard. However, there are still a few issues needing attention.	(No response required)

COMMENTS	RESPONSE
1) For the subgroup analyses on low and high burden groups, there are 6% missing data (without disease burden classification). But I haven't seen any sensitivity analysis on the missing data. What is the impact of missing data on the analysis results? Any potential bias? At least we'd like to see the patterns and characteristics of the patients without burden classification as compared to those with burden classification. May need to address the issue as a limitation in the discussion if missing is not at random.	The reason for missing volume data was primarily administrative: bureaucratic issues involved in retrospectively obtaining the images for analysis for sites. We do not think this relates to patient factors at all. Therefore we may argue in favour of the data being missing completely at random (MCAR).
2) In the analysis of the high-burden metastatic disease group (figue 2B), significant interaction was found. However, it is not clear whether the interaction term was dealt with in the analysis, and if yes, how it was dealt with. This question also applies to a few other analyses involving significant interations terms.	We have updated Section 2.5 of the Methods with further details.
3) Sample size calculation was never mentioned in the paper. I am aware this is a follow-up on the previous published paper but at least authors can mention the sample size and direct readers to previous papers or protocol.	We have highlighted in referencing the previous papers in Section 2.1 of the Methods that the sample size calculations can be found there. However, we can reiterate here if the Editor would find it helpful and space permits.
4) In statistical analysis section on page 11, it says "The proportional hazards assumption was tested", but with what method?	We have updated Section 2.5 of the Methods with further details.
**Requests from the editors:**	
In the Data Availability Statement (in the submission form) please include an appropriate contact email address for inquiries (this cannot be a study author).	mrcctu.datareleaserequest@ucl.ac.uk
Please remove the trial registration number from your title.	Removed
In your abstract ‘Methods and Findings’ please make the following changes:<br>* Please indicate the dates during which study enrollment and follow up occurred.<br>* Please include the duration of follow-up.<br>* Please clearly define the study outcomes.<br>* Please state that the analysis was intention-to-treat.<br>* Please include the important variables adjusted for in the analysis.<br>* Please state that there was no blinding to treatment allocation.<br>* Please include the important stratification factors adjusted for in the analysis.<br>* Please include a brief summary of adverse events.<br>* Please include a single sentence summary of the study’s main limitations at the end of the section (beginning ‘The main limitations of the study were…’, or similar).	We have updated the Abstract to capture this information. We were not sure whether the Abstract should mention limitations and seek editorial advice.

COMMENTS	RESPONSE
Please remove details of funding from the end of the abstract.	Done
At this stage, we ask that you include a short, non-technical Author Summary of your research to make findings accessible to a wide audience that includes both scientists and non-scientists. The Author Summary should immediately follow the Abstract in your revised manuscript. This text is subject to editorial change and should be distinct from the scientific abstract. Please see our author guidelines for more information.	Drafted and included in the manuscript.
Please update citations so that they are not superscript, are within square brackets, and precede punctuation.	We sought to implement the PLoS style in EndNote X9 which may not have worked. We have re-applied now the corresponding author has been moved to EndNote 20 and hope this works better.
Please provide the name of the relevant Institutional Review Board/s that provided ethics approval in the Methods.	This has been added.
Thank you for providing a CONSORT checklist. Please do the following:<br>* Update the checklist to use section names and paragraph numbers rather than page numbers (which change during the revision process).<br>* Cite the checklist in your methods (e.g. 'This trial is reported per the Consolidated Standards of Reporting Trials (CONSORT; see S1 checklist)' or similar).	This has been included.
Please report p values to 3 decimal places ($p<0.001$) (e.g. at lines 207 and 218, but please check throughout).	Done with original p-values added in footnotes.
Please define abbreviations used in your Tables and Figures (in the legend or in the figure/table itself where appropriate).	We have added a key to each Table and Figure. The abbreviations also appear in the overall abbreviation list.
Please provide the unadjusted comparisons as well as the adjusted comparisons in Table 2. Please also indicate factors adjusted for in the legend.	We have included these unadjusted comparisons in Table 2 but this was not part of the SAP, so we are a little uncomfortable presenting them.
Line numbering appears to disappear after page 17, please correct this to be continuous throughout the document.	We already see line numbering on every page so are not sure what action to take.
In your Discussion, please include a paragraph covering the strengths and limitations of the study.	We have added:<br>*"The trial has several strengths, including the randomised design, the large number of events for analysis, and recruitment from over 100 centres which adds to the generalisability of the results. Limitations are the changes in clinical practice since the trial started, particularly with regard to imaging techniques and systemic treatment."*
Author contributions and Competing interests can be removed from the manuscript as this information is captured as part of the submission form.	Removed

COMMENTS	RESPONSE
Please remove any formatting from your references (e.g. bold/italics) and only use et al. after listing the first six authors.	We hope we have now successfully implemented the correct style using EndNote 20.
**Requests from the authors:**	
n/a	We need to follow up our statement in the initial covering letter: *"We wish to be clear that the author list includes one person, Hassan Douis, who has not managed to complete their review of the manuscript. This person contributed in a way that qualifies for authorship and was included as an author on the first results from this trial in STAMPEDE. However, he has been on sabbatical for some months and is entirely non-contactable. We anticipate that he will return at the end of January, before the peer review process is completed, and agree to be named as an author. However, if he does not provide consent by that time, we will remove him from the author list. We have made this approach clear to him in an email which is waiting for him, and we hope this is an acceptable initial approach for the editorial office."*<br>Unfortunately, we have not received any communications from Hassan Douis since his sabbatical period must have ended. Therefore, we will have to remove him as an author. This will be tracked on the manuscript.
n/a	Please note that many of the authors struggled to complete the forms linked to them by PLoS Medicine. We think all have submitted them now. Please advise if they have not.<br>We think this is a problem common to all journals using the Editorial Manager system. We raise it to ensure that editorial teams for journals are aware and might be able to make the process easier for others in the future.
