# Supplementary material for: Radiotherapy to the prostate for men with metastatic prostate cancer in the UK and Switzerland: Long-term results from the STAMPEDE randomised controlled trial
Source: PLoS Med. 2022 Jun 7;19(6):e1003998. doi: 10.1371/journal.pmed.1003998 (PMC9173627; doi:10.1371/journal.pmed.1003998)
Supplement: Attachment — Submitted filename: Responses_2022-04-11_v1.pdf [file pmed.1003998.s017.pdf]

PMEDICINE-D-22-00022R2: COMMENTS FROM EDITORS AND REVIEWERS - AUTHOR RESPONSES
11-Apr-2022

JOURNAL COMMENT	AUTHOR RESPONSE
**Cover note**	
We ask every co-author listed on the manuscript to fill in a contributing author statement. If any of the co-authors have not filled in the statement, we will remind them to do so when the paper is revised. If all statements are not completed in a timely fashion this could hold up the re-review process. Should there be a problem getting one of your co-authors to fill in a statement we will be in contact. YOU MUST NOT ADD OR REMOVE AUTHORS UNLESS YOU HAVE ALERTED THE EDITOR HANDLING THE MANUSCRIPT TO THE CHANGE AND THEY SPECIFICALLY HAVE AGREED TO IT.	We have collected the forms with the exception of one person who has been on sabbatical and has not response. As previously discussed, we expect to follow the journal's unresponsive author protocol at this stage.
Please ensure that the paper adheres to the PLOS Data Availability Policy (see https://journals.plos.org/plosmedicine/s/data-availability), which requires that all data underlying the study's findings be provided in a repository or as Supporting Information. For data residing with a third party, authors are required to provide instructions with contact information for obtaining the data. PLOS journals do not allow statements supported by "data not shown" or "unpublished results." For such statements, authors must provide supporting data or cite public sources that include it.	We have included the data sharing statement. Data from clinical trials are available following the standard process of MRC CTU at UCL which is committed to supporting data sharing: https://www.mrcctu.ucl.ac.uk/our-research/other-research-policy/data-sharing/ This builds on Sydes et al (2015) Trials (10.1186/s13063-015-0604-6).
To enhance the reproducibility of your results, we recommend that you deposit your laboratory protocols in protocols.io, where a protocol can be assigned its own identifier (DOI) such that it can be cited independently in the future. Additionally, PLOS ONE offers an option to publish peer-reviewed clinical study protocols. Read more information on sharing protocols at https://plos.org/protocols/?utm_medium=editorial-email&utm_source=authorletters&utm_campaign=protocols	We included versions of the clinical trial protocol in the appendix and the protocol is available on the trial website.
Please review your reference list to ensure that it is complete and correct. If you have cited papers that have been retracted, please include the rationale for doing so in the manuscript text, or remove these references and replace them with relevant current references. Any changes to the reference list should be mentioned in the rebuttal letter that accompanies your revised manuscript.	Check – we believe this is okay.
Please note, when your manuscript is accepted, an uncorrected proof of your manuscript will be published online ahead of the final version, unless you've already opted out via the online submission form. If, for any reason, you do not want an earlier version of your manuscript published online or are unsure if you have already indicated as such, please let the journal staff know immediately at plosmedicine@plos.org.	We agree with this approach.

JOURNAL COMMENT	AUTHOR RESPONSE
If you have any questions in the meantime, please contact me or the journal staff on plosmedicine@plos.org.<br>We look forward to receiving the revised manuscript by Apr 08 2022 11:59PM.<br>Sincerely,<br>Callam Davidson,<br>Associate Editor<br>PLOS Medicine<br>plosmedicine.org	Appreciated
**Requests from Editors:**	
Please update your title to include the setting ('Radiotherapy to the prostate for men with metastatic prostate cancer in the UK and Switzerland: long-term results from the STAMPEDE randomised controlled trial').	Completed.
Data Availability Statement: Thank you for providing a contact email address (mrcctu.datareleaserequest@ucl.ac.uk). Please ensure this is included in your Data Availability Statement (via the submission form), as the current version recommends contacting the trial team (PLOS does not permit listing study authors as the point of contact for data requests).	This has been added.
In your abstract 'Methods and Findings' please make the following changes:	(see below)
* Please include the setting.	The country and the setting has been included in the Methods and the Abstract.
* Please include the length of follow up	Median follow-up is presented.
* Per our in-house style, please include a single sentence summary of the study's main limitations at the end of the section (beginning 'The main limitations of the study were…', or similar). See previous PLOS Medicine papers for examples.	We have changed in the Discussion from "Limitations are the changes in clinical practice since the trial started…" to "The main limitations of the study are the changes in clinical practice since the trial started…"
Please ensure the Abstract and Title are both updated in the submission form as well as the main paper (currently the two versions differ).	The updated versions have been copied into the system.
Please report p values to 3 decimal places throughout the document (P<0.001 should be the minimum value reported). Example at line 27 and in Figure 2A, but please check throughout.	It is our policy to present full p-values and should aim to do this in the version that will end up in our institutional repository. We would appreciate it if this change to house style could be done by the proofing team.
There is no need to include the original p values as footnotes.	(As above)
Line 35: Please update to 'indicating that it should be recommended as a standard of care' (applies also to concluding paragraph).	We have made this change to the Abstract.
Citations throughout should precede punctuation throughout.	We have made this change. (Perhaps this could have been done by the copy-editing team.)

JOURNAL COMMENT	AUTHOR RESPONSE
Your Author Summary should follow the format here: https://journals.plos.org/plosmedicine/s/revising-your-manuscript#loc-author-summary	We have drafted a new version.
Please update your CONSORT checklist to use section names and paragraph numbers rather than page numbers (which change during the revision process).	Done, assuming the paragraphs don't get combined in editing.
The name of the CONSORT checklist file also seems like it does not match the manuscript ('The Impact of a Community-Oriented Problem-Based Learning Curriculum Reform on the Quality of Primary Care Delivered by Graduates'). Please check.	This has been removed in v2.0. (Interestingly, this is hidden info was in the template you download from the CONSORT website!)
Thank you for presenting your unadjusted comparisons in Table 2. A line can be added to the Methods section noting that these comparisons were presented in response to a request from the handling editor and thus represents a change from the SAP.	Added: 'Unadjusted estimates of treatment effect are also presented.'
The Financial Disclosure can be removed from the main text, the relevant information should instead be captured in the submission form (answers will then be published as metadata).	This has been deleted from main manuscript.
Please remove the abbreviations list from the main text and instead define abbreviations on first use/in table and figure legends.	We have removed this. We hope it was useful for reviewers in the interim and wish more journals included an abbreviate list.
Line 88: Please update the citations here to [2, 3].	We believe we have done this.
When presenting hazard ratios in the Results, please consistently indicate whether these are adjusted or unadjusted ratios (e.g., Section 3.3, paragraph 1 and paragraph 2, HRs are reported differently despite both representing adjusted HRs).	We have updated in main draft and supplement. Have clarified that HRs reported in the text are from adjusted models.
Apologies if I missed it but I couldn't find an in-text reference for Figure S5?	Thank you for spotting this. We have added a cross-reference.
**Comments from Reviewers:**	
**Reviewer #1:**	
I would like to thank the authors for appropriately addressing the comments which I previously raised. I do not have any additional major comments. The authors should be congratulated on this important contribution.	Appreciated
Of note, Table S5 appears to be currently formatted in such a way that the right portion of the table is cut off and not visible in either the PDF or the Word document. I would recommend that the orientation of this page be changed to landscape to allow for the entire table to be viewed.	We have fixed this.
**Reviewer #2:**	
The authors made several improvements to the document based on the reviewers comments, I have no further issues to raise.	No response required

JOURNAL COMMENT	AUTHOR RESPONSE
**Reviewer #3:**	
Thanks authors for their effort to improve the manuscript. I am satisfied with the response and revision. No further issues needing attention.	Appreciated
